# Therapeutic mechanisms of the medicine and food homology formula Xiao-Ke-Yin on glucolipid metabolic dysfunction revealed by transcriptomics, metabolomics and microbiomics in mice

**DOI:** 10.1186/s13020-023-00752-6

**Published:** 2023-05-18

**Authors:** Mei Li, Ding Cheng, Chuan Peng, Yujiao Huang, Jie Geng, Guangrui Huang, Ting Wang, Anlong Xu

**Affiliations:** 1 School of Life Sciences, Beijing University of Chinese Medicine, Beijing, China; 2 Chengdu University of Traditional Chinese Medicine, Chengdu, China; 3 Beijing Research Institute of Chinese Medicine, Beijing University of Chinese Medicine, Beijing, China

**Keywords:** Xiao-Ke-Yin, *db/db* mice, Glucolipid metabolism, Transcriptomics, Gut microbiota, Metabolomics

## Abstract

**Background:**

In recent decades, the prevalence of metabolic diseases, particularly diabetes, hyperlipidemia, obesity, and non-alcoholic fatty liver disease (NAFLD), has increased dramatically, causing great public health and economic burdens worldwide. Traditional Chinese medicine (TCM) serves as an effective therapeutic choice. Xiao-Ke-Yin (XKY) is a medicine and food homology TCM formula consisting of nine “medicine and food homology” herbs and is used to ameliorate metabolic diseases, such as insulin resistance, diabetes, hyperlipidemia and NAFLD. However, despite its therapeutic potential in metabolic disorders, the underlying mechanisms of this TCM remain unclear. This study aimed to evaluate the therapeutic effectiveness of XKY on glucolipid metabolism dysfunction and explore the potential mechanisms in *db/db* mice.

**Methods:**

To verify the effects of XKY, *db/db* mice were treated with different concentrations of XKY (5.2, 2.6 and 1.3 g/kg/d) and metformin (0.2 g/kg/d, a hypoglycemic positive control) for 6 weeks, respectively. During this study, we detected the body weight (BW) and fasting blood glucose (FBG), oral glucose tolerance test (OGTT), insulin tolerance test (ITT), daily food intake and water intake. At the end of the animal experiment, blood samples, feces, liver and intestinal tissue of mice in all groups were collected. The potential mechanisms were investigated by using hepatic RNA sequencing, 16 S rRNA sequencing of the gut microbiota and metabolomics analysis.

**Results:**

XKY efficiently mitigated hyperglycemia, IR, hyperlipidemia, inflammation and hepatic pathological injury in a dose dependent manner. Mechanistically, hepatic transcriptomic analysis showed that XKY treatment significantly reversed the upregulated cholesterol biosynthesis which was further confirmed by RT-qPCR. Additionally, XKY administration maintained intestinal epithelial homeostasis, modulated gut microbiota dysbiosis, and regulated its metabolites. In particular, XKY decreased secondary bile acid producing bacteria (*Clostridia* and *Lachnospircaeae*) and lowered fecal secondary bile acid (lithocholic acid (LCA) and deoxycholic acid (DCA)) levels to promote hepatic bile acid synthesis by inhibiting the LCA/DCA-FXR-FGF15 signalling pathway. Furthermore, XKY regulated amino acid metabolism including arginine biosynthesis, alanine, aspartate and glutamate metabolism, phenylalanine, tyrosine and tryptophan biosynthesis, and tryptophan metabolism likely by increasing *Bacilli*, *Lactobacillaceae* and *Lactobacillus*, and decreasing *Clostridia*, *Lachnospircaeae*, *Tannerellaceae* and *Parabacteroides* abundances.

**Conclusion:**

Taken together, our findings demonstrate that XKY is a promising “medicine food homology” formula for ameliorating glucolipid metabolism and reveal that the therapeutic effects of XKY may due to its downregulation of hepatic cholesterol biosynthesis and modulation of the dysbiosis of the gut microbiota and metabolites.

**Supplementary Information:**

The online version contains supplementary material available at 10.1186/s13020-023-00752-6.

## Introduction

In recent decades, the prevalence of metabolic diseases, particularly diabetes, hyperlipidemia, obesity and non-alcoholic fatty liver disease (NAFLD), has increased dramatically, thereby causing great public health and economic burden worldwide [1, 2]. As a consequence of the pandemic spread of obesity, the global prevalence of NAFLD is approximately 25%, making it the leading cause of chronic liver disease [3, 4]. According to the data from the International Diabetes Federation, the incidence of diabetes mellitus continues to grow and will reach 643 million by 2030 and 783 million by 2045 [5]. Indeed, due to the similar pathogenesis, NAFLD patients are often clinically diagnosed associated with other metabolic diseases, including obesity, insulin resistance (IR), hyperlipidemia or (and) type 2 diabetes mellitus (T2DM), clinically [2, 3, 6, 7].

Metabolic diseases such as T2DM and NAFLD are generally characterized by a dysregulated glucolipid metabolism, IR, chronic low-grade inflammation, and diffuse hepatic fat accumulation [6, 8]. Although the underlying molecular mechanisms are not fully understood, recent studies are continuously adding new evidence that gut microbiota dysbiosis contributes to the onset and progression of metabolic diseases and can be exploited for its therapy [9]. For instance, the relative abundance of *Lactobacillus* is lower in both NAFLD patients and diabetic rats than in healthy subjects, and ingestion of *Lactobacillus* reduces total cholesterol (TC) and triglyceride (TG) levels to ameliorate non-alcoholic steatosis, suggesting that *Lactobacillus* provides a useful strategy for the treatment of NAFLD [10, 11]. Importantly, the gut microbiota is a virtual endocrine organ producing metabolites, including bile acids (BAs), lipopolysaccharides (LPS), amino acids (AAs) and short-chain fatty acids (SFAs), and the altered gut microbiota disrupts intestinal barrier integrity allowing for the subsequent translocation of bacterial metabolites [12–14]. Gut microbiota-derived metabolites interact with the host at local or distal target organs such as liver and brain, and regulate metabolic phenotypes contributing to metabolic disease development [12, 13, 15].

Considerable progress in therapeutic strategies including healthy dietary habits [16], appropriate physical exercise [17], prebiotics [18], functional amino acid ingestion [19], and the use of pharmacological drugs [7] for metabolic diseases has occurred. Traditional Chinese medicine (TCM) has been applied to the treatment of metabolic diseases for many years, and increasing evidence shows that TCM improves metabolic diseases via the modulation of the gut microbiota and its metabolites [20]. Some edible herbs offer benefits beyond nutrition and are thus generally used as medicine as well as functional foods in people’s daily life [21]. These herbs are also called “medicine food homology” or “drug homologous food” herbs are widely accepted to possess various ingredients and functions with low toxicity and side effects, indicating a promising alternative therapy choice [22]. In clinical practice, TCMs, including “medicine food homology” herbs, are generally combined with the guide of TCM theories to create a formula that could strengthen their effects. For instance, a previous study showed that a diet herbal formula PLCP, including four herbs (*Puerariae radix*, Lycium barbarum, *Crataegus pinnatifida*, and *Polygonati rhizome*), protected against IR and NAFLD in high fat and high fructose diet-fed mice [23]. In TCM theory, patients with glucolipid metabolism dysfunction such as insulin resistance, or T2DM with NAFLD are characterized by phlegm-dampness, spleen deficiency and yin deficiency with effulgent fire. XKY (Xiao-Ke-Yin, Patent number: 201610367493.1), a TCM formula consisting of nine “medicine food homology” herbs (*Dioscorea oppositifolia L*, *Polygonatum sibiricum*, *Rhizoma Polygonati Odorati*, *Folium Mori, Crataegus pinnatifida Bunge*, *Rhizoma Phragmitis*, *Angelica sinensis (Oliv.) Diels.*, *Gardenia jasminoides* Ellis and *Pueraria lobata* (Willd.) Ohwi), was originally designed by Professor Ting Wang in Beijing University of Traditional Chinese Medicine to ameliorate glucolipid metabolic dysfunction-associated metabolic diseases, with the guidance of TCM theory. The “Monarch (Jun)” medicine in XKY is *Dioscorea oppositifolia* L. The “Minister (Chen)” medicines in XKY are *Polygonatum sibiricum*, *Pueraria lobata* (Willd.) Ohwi), *Folium Mori*, *Crataegus pinnatifida Bunge*, *Rhizoma Phragmitis* and *Angelica sinensis* (*Oliv.*) Diels. The “Adjuvant (Zuo)” medicines in XKY are *Gardenia jasminoides* Ellis and *Rhizoma Polygonati Odorati*. The whole formula has the functions of nourishing spleen and tonifying Qi, nourishing Yin and clearing Heat, disinhibiting Dampness and resolving Phlegm. Studies have demonstrated the therapeutic effects of these individual herbs on metabolic diseases, and also XKY showed a potential protective role in metabolic disorder patients in our clinical trial (ChiCTR2000040574) supported by National Major Science and Technology Project (Grant number: 2019YFC1710100). Thus, we hypothesized that XKY has promising therapeutic benefits, but its underlying mechanisms remain largely unknown.

The present study is aimed to evaluate the therapeutic effectiveness and the underlying mechanisms of XKY in *db/db* mice. We explored the efficacy of XKY on hyperglycemia, IR and inflammation, and assessed its capability to regulate hepatic glucolipid metabolism and gut microbiota as well as its metabolites. Our findings indicate that XKY is a promising TCM formula for the treatment of metabolic diseases.

## Materials and methods

### Preparation of XKY Formula

XKY is a TCM formula containing nine “medicine food homology” natural herbs, including *Dioscorea oppositifolia L*, *Polygonatum sibiricum*, Rhizoma Polygonati Odorati, *Folium Mori*, *Crataegus pinnatifida Bunge*, *Rhizoma Phragmitis*, Angelica sinensis (Oliv.) Diels., Gardenia jasminoides Ellis and *Pueraria lobata (Willd.)* Ohwi, with the China patent number of 201610367493.1. All of the herbs were purchased from Beijing Kangrentang Pharmaceutical Co., Ltd. and authenticated by Professor Ting Wang (Beijing University of Chinese Medicine, Being, China). XKY extract was prepared by Jiangxi Baoli Pharmaceutical Co., Ltd. (Beijing, China) under a strict GMP production process as follows. All herbs material were precisely weighed and were soaked together in a 10-time volume double distilled water for 30 min before being boiled. The herbs were decocted for 90 min for the first time and the decoction was centrifuged at 4000 for 10 min to obtain supernatants. Subsequently, the mixture herbs were boiled a second time with an additional 8-time volume double distilled water for 60 min and centrifuged at 4000 for 10 min to obtain the second decoction supernatants. Then the supernatants of the first and second decoction were mixed and concentrated under reduced pressure at 60–80 ℃ to obtain XKY extract. The extract was shattered and filtered through an 80-mesh sieve. Finally, the XKY extract was used in this study as well as in our clinical trial (20 g/d/person).

### Analysis of the XKY chemical profile

To analyze the constituents of XKY, we carried out a UPLC-QE-MS assay. First, we used fluid initial solution (methanol: water = 4:1) to dilute XKY. After vortexing and homogenate, the solution was centrifuged at 12,000 rpm for 15 min. Then, the supernatant was filtered with a 0.22 μm filter. Subsequently, the prepared sample was analyzed with an Agilent ultrahigh-performance liquid chromatography 1290 UPLC-Q Exactive Focus system with a Waters UPLC BEH C18 column (1.7 μm 2.1*100 mm) at 55 °C. A 5 µL sample was injected, and the flow rate was set at 0.5 mL/min. The mobile phase consisted of 0.1% formic acid in water (A) and 0.1% formic acid in acetonitrile (B). The multistep linear elution gradient program was as follows: 0–11 min, 85 − 25% A; 11–12 min, 25 − 2% A; 12–14 min, 2–2% A; 14-14.1 min, 2–85% A; and 14.1–16 min, 85–85% A. To obtain the MS and MS/MS data, we used a Q Exactive Focus mass spectrometer coupled with Xcalibur software based on IDA acquisition mode. The following parameters were applied: sheath gas flow rate, 45 psi; aux gas flow rate, 15 psi; capillary temperature, 400 °C; full ms resolution, 70,000; MS/MS resolution, 17,500; collision energy, 15/30/45 in NCE mode; and spray voltage, 4.0 kV (ESI+) or − 3.6 kV (ESI-). The mass spectrometry fragments of the tested sample XKY were matched with the in-house secondary mass spectrometry database (Shanghai BIOTREE Biotech Co., Ltd) [24].

### Animals and experimental design

In this study, male C57BL/KSJ-*db/db* mice (*db/db*, 3–4 weeks) and male C57BL/KSJ-wt/wt mice of the same age were purchased from Nanjing Biomedical Research Institute of Nanjing University (Nanjing, China) (license nu mber: SCXK (SU) 2016 0011). After arrival, all mice were housed at a temperature of 25 ± 2 °C, relative humidity of 50 ± 5% and a 12/12-hour day-night cycle under specific pathogen-free conditions. All mice had free access to food and water. Following 2 weeks of adaptive breeding, mice were randomly divided into five groups (nine mice in each group): the model group (Model), metformin group (MET), XKY high-dose group (XKY-H), XKY middle-dose group (XKY-M) and XKY low-dose group (XKY-L). Nine C57BL/KSJ-wt/wt mice at the same age were used as the normal control group (NC). The MET group was orally administrated with metformin (Sino-American Shanghai Squibb Pharmaceutical Ltd, Shanghai China) at a dose of 0.2 g/kg/d and served as the positive control to evaluate hypoglycemic effectiveness. The XKY-H, XKY-M and XKY-L groups were orally administrated with XKY at doses of 5.2 g/kg/d, 2.6 g/kg/d and 1.3 g/kg/d, respectively. The middle dose of XKY was equivalent to approximately a dose of 20 g/d/person in humans according to an extrapolation performed using the body surface area normalization method as follows: (X mg/kg/d * 70 kg * 0.0026)/20 g = 9.1*X mg/kg/d, where X mg/kg/d is the dose of human. The high dose is twice of the middle dose and the low dose is half of the middle dose.

During this study, BW and FBG were monitored weekly. The oral glucose tolerance test (OGTT), insulin tolerance test (ITT), daily food intake and water intake were measured after a 6-week treatment. At the end of the animal experiment, blood samples, feces, livers and intestine tissues of mice in all groups were collected after 12 h of food deprivation. This study was conducted in strict accordance with the Guide for Care and Use of Laboratory Animals of Beijing University of Chinese Medicine (License number: BUCM-4-2021040108-2121).

### Measurement of FBG, OGTT and ITT levels

In this study, a blood glucose meter (Ultra, American) was used to measure the blood glucose level in tail vein blood. We measured FBG when mice were fasted for 12 h overnight. For OGTT, mice were fasted for 12 h overnight, and then the blood glucose levels were detected before and at 30, 60 and 120 min after administering 50% oral glucose (2 g/kg, BW). For ITT, mice were fasted for 4 h, and then the blood glucose levels were detected before and at 30, 60 and 120 min after subcutaneous injection of insulin (0.75 UI/kg, BW). The area under the curve (AUC) was calculated as follows: OGTT-_AUC_/ITT‐_AUC_ = 0.5 × (Bg 0 min + Bg 30 min)/2 + 0.5 × (Bg 30 min + Bg 60 min)/2 + 1 × (Bg 60 min + Bg 120 min)/2, where Bg is the blood glucose level at each time point.

### Hematoxylin and eosin (HE), periodic acid-Schiff (PAS) and oil red O stainings

For hematoxylin and eosin (HE) and periodic acid-Schiff (PAS) staining, freshly collected liver and small intestine tissues were fixed in 4% paraformaldehyde (Solarbio, Beijing, China) and then embedded in paraffin. Subsequently, liver and small intestine slides were stained with HE according to the manufacturer’s instructions (Solarbio, Beijing, China). Hepatic PAS staining was carried out using a PAS staining kit following the manufacturer’s instructions (Solarbio, Beijing, China). For hepatic Oil Red O staining, fresh frozen liver tissues were embedded in OCT. After preparing frozen sections, we used an Oil Red O staining solution kit (Solarbio, Beijing, China) to stain the sections following the manufacturer’s instructions.

### Measurement of serum insulin, FFA, TNF-α, IL-6, ALT, AST, TC, and TG levels and hepatic ALT, AST, TC and TG levels

We used ELISA kits (Cusabio, Wuhan, China) to measure serum levels of fasting insulin, TNF-α and IL‐6 according to the manufacturer’s instructions. Homeostasis assessment of insulin resistance (HOMA-IR) was calculated as follows: HOMA-IR = fasting insulin (mIU/L) × FBG (mmol/L)/22.5. The levels of FFA, TC, TG, AST and ALT in the liver tissues and serum were detected using FFA, TC, TG, AST and ALT kits (Nanjing Jiancheng Bioengineering Institute, Nanjing, China) following the manufacturer’s instructions.

### Liver transcriptome sequencing

Total RNA was extracted from the liver tissues of the NC, model and XKY-H groups, and the RNA integrity was detected using the RNA Nano 6000 Assay Kit of the Bioanalyzer 2100 system (Agilent Technologies, CA, USA). Subsequently, library preparation was carried out using the total RNA, and the library quality was determined using the Agilent Bioanalyzer 2100 system. Then, the library preparations were sequenced on an Illumina NovaSeq platform by Novogene Biotechnology Co., Ltd. Finally, raw data were produced, and we removed reads containing adapters and poly-N and low-quality reads from the raw data to obtain clean data for all downstream analyses. The data were analyzed on Novomagic, a free online platform for data analysis (https://magic.novogene.com). Genes between two groups with FDR < 0.05 and |logFC| > 0.5 were considered differentially expressed genes (DEGs). For functional enrichment analysis, DEGs were subjected to the DAVID online tool (https://david.ncifcrf.gov/), and the significant enrichment of DEGs was determined by a *P*-value < 0.05. The original raw RNA sequencing data were deposited into the NCBI database (accession number: PRJNA889248).

### Real-time qPCR

For RT-qPCR, total RNA of tissue was extracted using a SteadyPure Universal RNA Extraction Kit (Accurate Biology, Beijing, China). After concentration measurement, cDNA was synthesized using the Evo M-MLV RT Premix for qPCR (Accurate Biology, Beijing, China). Real-time PCR was performed using the SYBR^®^ Green Premix Pro Taq HS qPCR Kit (Accurate Biology, Beijing, China) in an Applied Biosystems 7500 Real‐time PCR Instrument. The relative mRNA levels were normalized to β-Actin. The ∆∆Ct method was used to calculate the relative quantitation. All primers used in this study were listed in Additional file 1: Table S1.

### Immunohistochemical (IHC) staining and analysis

IHC staining was carried out as described previously [25]. Briefly, small intestine and liver paraffin tissues were sectioned into 5-µm slices. After incubating at 65 °C for 60 min, the slices were dewaxed and rehydrated by xylene and gradient ethanol. Then, 3% hydrogen peroxide and sodium citrate were used to block endogenous peroxidase activity and for epitope retrieval, respectively. After blocking, the slides were incubated with primary antibodies against ZO-1 (Proteintech, Wuhan, China), claudin-1 (Abcam, Cambridge, MA, USA), and CYP7A1 (Bioss, Beijing, China) overnight at 4 °C. On the second day, slides were incubated with the HRP-labeled anti-rabbit IgG secondary antibody for 30 min at room temperature. Subsequently, the sections were stained with DAB according to the manufacturer’s instructions (ZSGB-BIO, Beijing, China). Finally, the slides were counterstained with hematoxylin and dehydrated with an ethanol gradient. ImageJ was used to evaluate the immunostaining.

### Western blotting

For the Western blotting assay, frozen tissues were washed with ice-cold PBS, and the protein was extracted by a total extraction kit (KeyGEN, Nanjing, China). Equal amounts of proteins were separated by SDS‒PAGE and transferred to PVDF membranes (Millipore, County Cork, Ireland). Then, the membranes were subsequently blocked with QuickBlock™ Blocking Buffer for Western blot (Beyotime Biotechnology, Shanghai, China) at room temperature for 10 min and incubated with the indicated primary antibodies overnight at 4 °C. Next, the protein bands were incubated with secondary antibodies (Cell Signaling Technology, Danvers, MA, USA) at room temperature for 60 min. Then, the protein bands were detected and analyzed with an enhanced chemiluminescence kit (Solarbio, Beijing, China) using a Bio-Rad ChemiDoc XRS + Imaging System. The primary antibodies used were as follows: rabbit anti-β-Actin (Cell Signaling Technology, Danvers, MA, USA), rabbit anti-ZO-1 (Abcam, Cambridge, MA, USA), rabbit anti-Claudin-1 (Abcam, Cambridge, MA, USA), mouse anti-FXR (Santa Cruz, Shanghai, China), and rabbit anti-FGF15 (Abcam, Cambridge, MA, USA).

### Gut microbiota analysis

For gut microbiota analysis, fresh fecal samples were collected from the NC, Model and XKY-H group mice at the end of the animal experiment. After DNA extraction, the DNA extract was checked on a 1% agarose gel, and the DNA concentration and purity were detected using a NanoDrop 2000. The primers 338 F (5′-ACTCCTACGGGAGGCAGCAG‐3′) and 806R (5′‐GGACTACHVGGGTWTCTAAT‐3′) were used for PCR on an ABI GeneAmp^®^ 9700 PCR thermocycler (ABI, CA, USA) to amplify the V3-V4 of the bacterial 16 S rRNA gene. After the library was qualified, the Illumina MiSeq PE300 platform was used for 16 S rRNA sequencing according to the standard protocols by Majorbio Bio-Pharm Technology Co. Ltd. (Shanghai, China). The data were analyzed on the Majorbio Cloud Platform (www.majorbio.com). The original 16 S rDNA sequencing raw data were deposited into the NCBI database (accession number: PRJNA887907).

### Untargeted metabolomics analysis

Fresh fecal samples were collected from the NC, Model and XKY-H groups at the end of the animal experiment and subsequently sent to Majorbio Biotech Co., Ltd. for untargeted metabolomics analysis. First, 50 mg of feces from each sample was accurately weighed and dissolved in a 400 µL methanol: water (4:1, v/v) solution to extract the metabolites. L-2-chlorophenylalanin at a concentration of 0.02 mg/mL was used as the internal standard, and quality control samples (QCs) were used for system conditioning and quality control. A Thermo UHPLC system equipped with an ACQUITY UPLC HSS T3 (100 mm × 2.1 mm i.d., 1.8 μm; Waters, Milford, USA) was used to perform chromatographic separation. The mobile phases consisted of 0.1% formic acid in 95:5 water/acetonitrile (v/v) (A) and 0.1% formic acid in 47.5:47.5:5 acetonitrile/isopropanol/water (v/v/v) (B). The solvent gradient changed according to the following conditions: 0 to 0.1 min, 0% B to 5% B; 0.1 to 2 min, 5% B to 25% B; 2 to 9 min, 25% B to 100% B; 9 to 13 min, 100% B to 100% B; 13 to 13.1 min, 100% B to 0% B; and 13.1 to 16 min, 0% B to 0% B for equilibrating the systems. A 2 µL sample was injected, and the flow rate was set as 0.4 mL/min. and the column temperature was maintained at 40 °C.

The mass spectrometric data was collected using a Thermo UHPLC-Q Exactive mass spectrometer equipped with an electrospray ionization (ESI) source operating in either positive or negative ion mode. The detection was carried out over a mass range of 70–1050 m/z. The optimal conditions were set as follows: ion-spray voltage floating (ISVF), 2800 V in negative mode and 3500 V in positive mode; sheath gas flow rate, 40 psi; aux gas flow rate, 10 psi; heater temperature, 400 °C; and normalized collision energy, 20–40–60 V rolling for MS/MS. Full MS resolution was 70,000, and MS/MS resolution was 17,500.

After UPLC-MS analyses, Progenesis QI v2.3 (Nonlinear Dynamics, Waters, USA) was used for peak detection and alignment. The data matrix that consisted of the retention time (RT), mass-to-charge ratio (m/z) values, and peak intensity were obtained. Mass spectra of the metabolic features were identified by using the accurate mass, MS/MS fragment spectra and isotope ratio difference with searching in reliable biochemical databases such as the Human metabolome database (HMDB) (http://www.hmdb.ca/) and Metlin database (https://metlin.scripps.edu/). Concretely, the mass tolerance between the measured m/z values and the exact mass of the components of interest was ± 10 ppm. In this study, only peaks presenting a non-zero value for at least 80% were retained and the minimum metabolite value was used to replace missing values in which the metabolite levels fell below the lower limit of quantitation in the original data. The median normalization method followed by generalized log transformation was applied to identify significant differences in metabolite levels between comparable groups. The data were analyzed on the Majorbio Cloud Platform (www.majorbio.com).

### Statistical analysis

Data were expressed as means ± standard deviation with GraphPad Prism software (version 8.0). For data with more than two groups, analysis was conducted by one-way ANOVA. Statistical Package for Social Sciences (SPSS) software (version 23.0) was used for statistical analysis in this study. *P* < 0.05 (*P < 0.05, **P < 0.01, ***P < 0.001) was considered statistically significant.

## Results

### Identification of the chemical composition of XKY

**Fig. 1:**
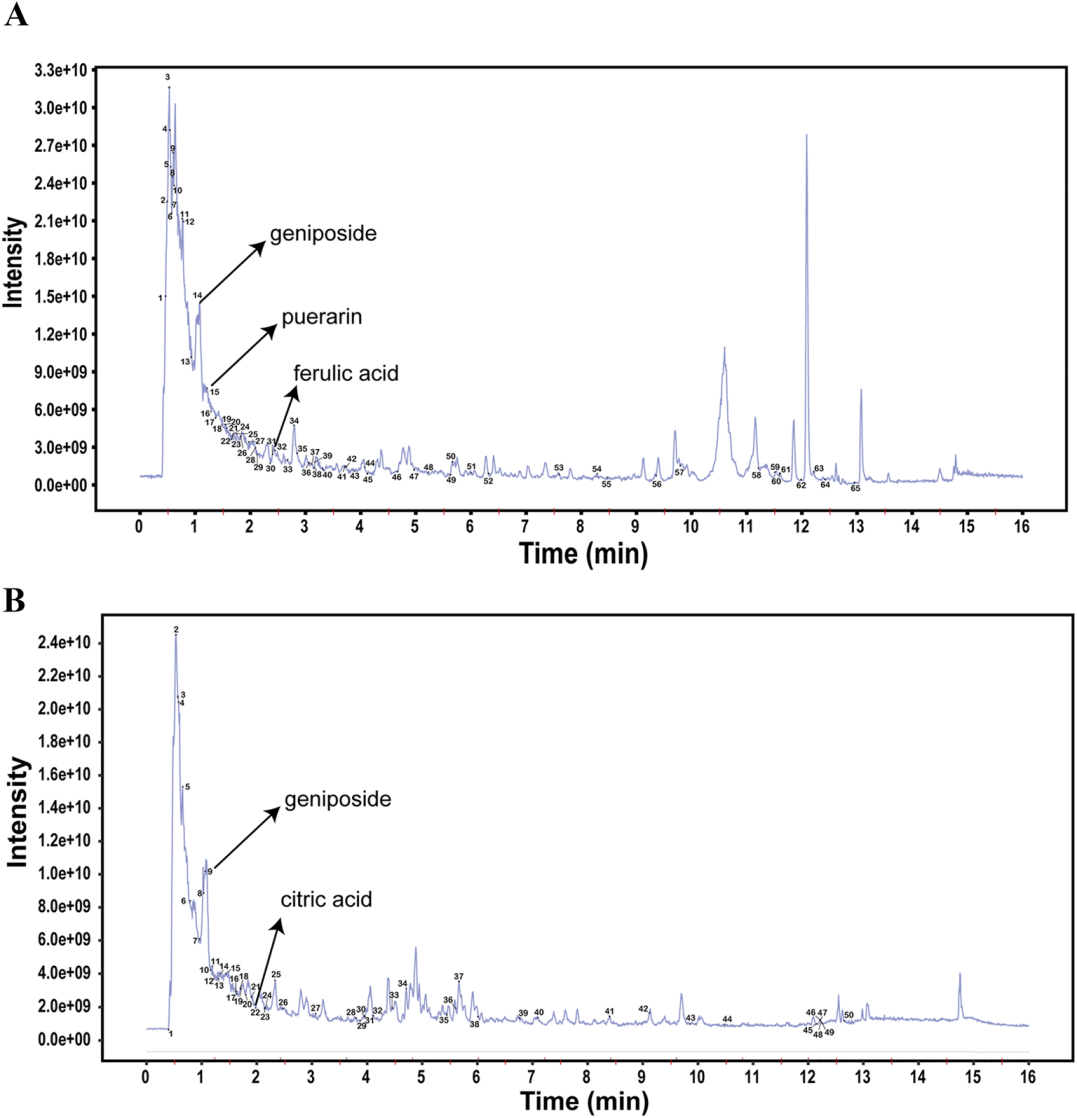
Total ion chromatogram of UPLC-QE-MS analysis of XKY in ESI + mode (**A**) and in ESI- mode (**B**)

To identify the constituents of XKY, a UPLC-QE-MS assay was performed. As shown in Fig. 1A and B, 65 and 50 chemicals were identified in ESI + mode and ESI- mode respectively. These chemicals included some marker ingredients (geniposide, citric acid, puerarin and ferulic acid) of the individual herbs in XKY according to the China Pharmacopoeia. Detailed information on the identified chemicals was shown in  Additional file 1: Tables S2 and S3.

### XKY alleviates hyperglycemia and insulin resistance in *db/db* mice

**Fig. 2:**
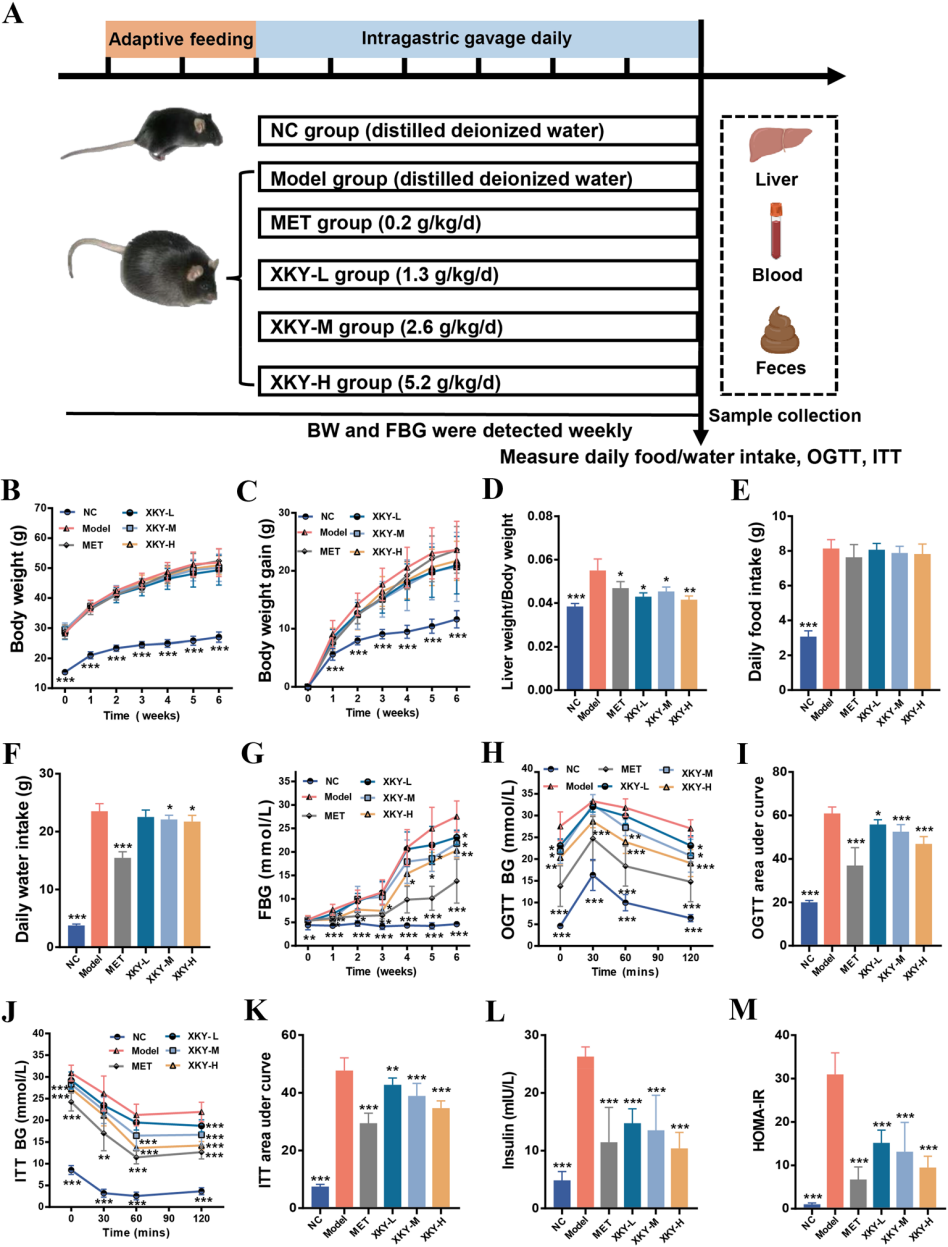
Effects of XKY on hyperglycemia and insulin resistance in *db/db* mice. **A** Schematic diagram of the animal experiment. **B** Body weight, **C** body weight gain, **D** liver weight/body weight, **E** daily food intake, **F** daily water intake, **G** FBG, **H** curve of OGTT, **I** OGTT area under curve, **J** curve of ITT, **K** ITT area under curve, **L** insulin and **M** HOMA-IR in NC, Model, MET, XKY-H, XKY-M and XKY-L groups. Data were shown as means ± SD (*n* = 6–9, *P < 0.05, **P < 0.01, ***P < 0.001 vs. the Model group)

To verify the effects of XKY in vivo, *db/db* mice were treated with Metformin (MET) (0.2 g/kg/d, a hypoglycemic positive control), high dose of XKY (XKY-H) (5.2 g/kg/d), moderate dose of XKY (XKY-M) (2.6 g/kg/d) or a low dose of XKY (XKY-L) (1.3 g/kg/d) for 6 weeks (Fig. 2A). As shown in Fig. 2B and C, the body weight (BW) and BW gain of the Model group were significantly increased compared with those of the NC group, but there was no significant difference between the treatment groups and the Model group. Interestingly, the liver weight index (liver weight/BW) was significantly higher in the Model group and lower in the MET and XKY treatment groups (Fig. 2D). In addition, the daily food intake also showed no obvious differences between the Model group and the treatment groups, while the daily water intake increased in Model group and decreased in the MET, XKY-H, and XKY-M groups (Fig. 2E and F), indicating an improvement in polydipsia symptoms. After XKY-H, XKY-M, XKY-L and MET treatment, decreased FBG levels were obviously observed in the Model group compared with the NC group, suggesting that XKY exerts a hypoglycemic effect in a dose-dependent manner (Fig. 2G).

Since the pathogenesis of metabolic diseases is closely associated with IR, we conducted OGTT and ITT after the 6 weeks of treatment. As shown in Fig. 2H, during the OGTT, the blood glucose values peaked at 30 min after glucose administration and then declined in all tested groups. Figure 2 J depicts the glucose levels during ITT, and the blood glucose values decreased in all tested groups upon insulin injection. The OGTT and ITT curves showed that the MET and XKY treatment groups presented lower glucose levels than the Model group at the same time point, especially at 120 min after glucose administration or insulin injection, indicating that MET and XKY treatments significantly improved glucose and insulin tolerance in *db/db* mice. XKY-H presented the most effective group among the three doses. OGTT-_AUC_ and ITT‐_AUC_ were dramatically increased in the Model group compared with the NC group, while all the treatment groups exhibited a substantial reduction (Fig. 2I and K). Notably, the XKY-H group showed a lower OGTT‐_AUC_ and ITT‐_AUC_ than the XKY-M and XKY-L groups (Fig. 2I and K).

Furthermore, we found that the insulin level of the Model group was markedly elevated compared with that of the NC group, whereas both XKY and MET treatment significantly decreased insulin levels (Fig. 2L). Importantly, the HOMA-IR index of the Model group was obviously increased compared with that of the NC group, while XKY reduced the HOMA‐IR index in a dose‐dependent manner (Fig. 2M). Conclusively, these data provide strong evidence that XKY can improve glycaemic control and IR in *db/db* mice.

### XKY ameliorated liver steatosis, hyperlipidemia and inflammation in *db/db* mice

**Fig. 3:**
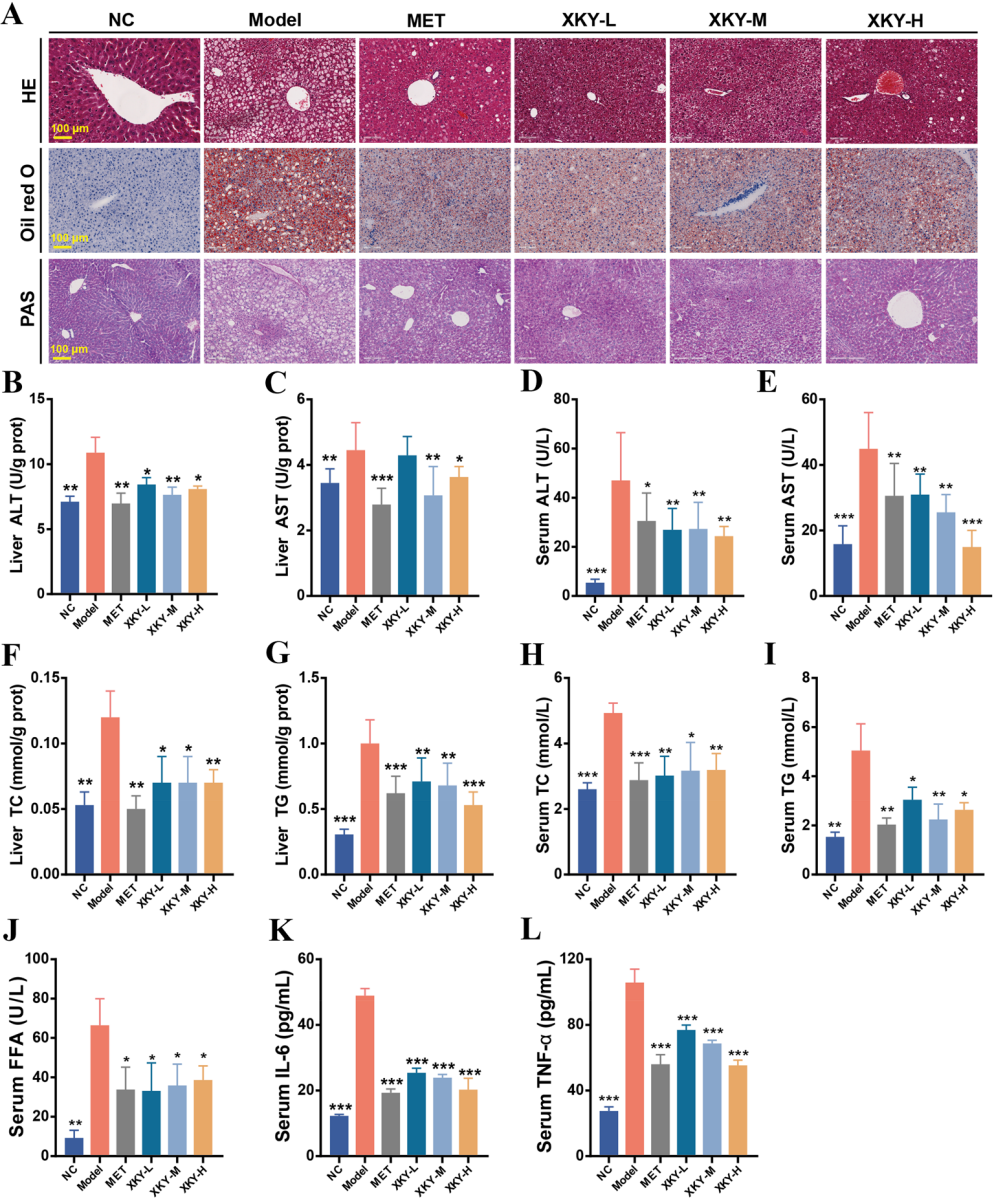
Effects of XKY on glucolipid metabolism and inflammatory cytokines in *db/db* mice. **A** Representative images of hepatic histopathological examination, Oil Red O staining and PAS staining in NC, Model, MET, XKY-H, XKY-M and XKY-L groups. Representative images were presented. Scale bar represents 100 μm. Levels of liver ALT (**B**), liver AST (**C**), serum ALT (**D**), serum AST (**E**), liver TC (**F**), liver TG (**G**), serum TC (**H**), serum TG (**I**), serum FFA (**J**), serum IL-6 (**K**) and serum TNF-α (**L**) in NC, Model, MET, XKY-H, XKY-M and XKY-L groups. Data were shown as means ± SD (*n* = 6, *P < 0.05, **P < 0.01, ***P < 0.001 vs. the Model group)

The liver plays a central role in regulating glucose and lipid metabolism. Next, we investigated the effect of XKY on the histological and glucolipid metabolic characteristics of the liver in *db/db* mice. As shown in Fig. 3A, HE staining and Oil Red O staining demonstrated obvious hepatic morphology injury and steatosis with increased lipid droplets in the Model group which were mitigated by MET and different doses of XKY treatments. In addition, we observed significantly lower glycogen deposition in the Model group than the NC group according to hepatic PAS staining, whereas hepatic glycogen accumulation was increased after XKY and MET administration (Fig. 3A). ALT and AST levels in liver and serum were increased in the Model group and decreased following MET and XKY treatments, suggesting that XKY and MET may ameliorate abnormal liver function in *db/db* mice (Fig. 3B–E). In addition, the levels of TC and TG in the liver and serum as well as the level of serum FFA in the Model group were robustly elevated, which were reversed by XKY and MET treatments, indicating that XKY and MET improved the lipid profile in *db/db* mice (Fig. 3F–I). Metabolic diseases are generally accompanied by chronic low-grade inflammation. As expected, IL-6 and TNF-α were increased in the Model group and dramatically decreased in the XKY and MET groups (Fig. 3L and M). Therefore, these results reveal the protective roles of XKY on hepatic morphology recovery, glucose and lipid metabolism and inflammation in *db/db* mice.

### XKY altered the hepatic transcriptome and downregulated the genes involved in cholesterol synthesis

**Fig. 4:**
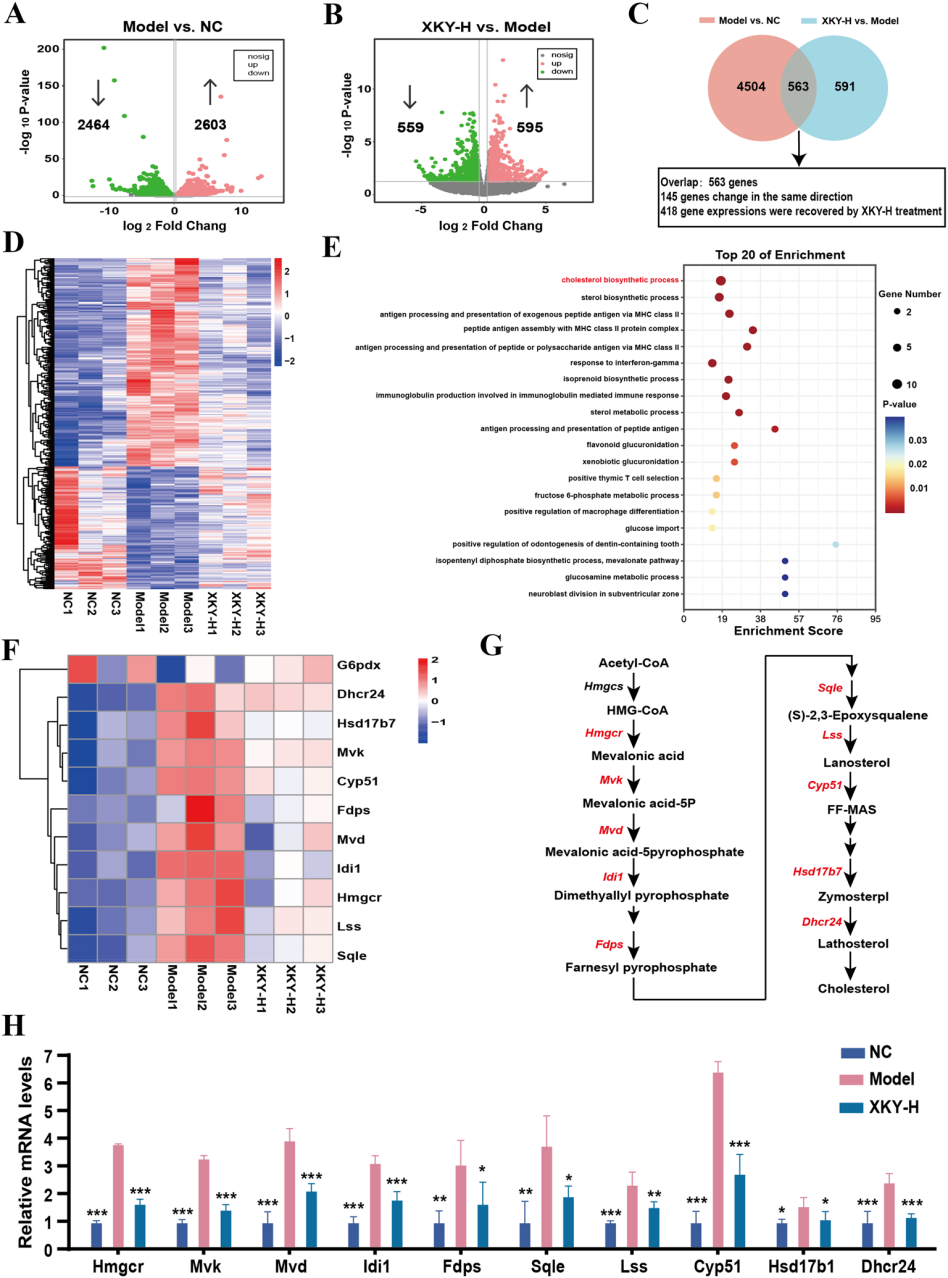
Effects of XKY on transcriptomic profiling of the liver in *db/db* mice. Volcano plot of the DEGs of the NC group vs. Model group (**A**), XKY-H group vs. Model group (**B**). Red color, green color, and grey color points indicated the significantly upregulated genes, downregulated genes and not significantly changed genes respectively. The gray dashed horizontal line indicated the FDR < 0.05, and the gray perpendicular dotted line indicated the |log_2_FC| > 0.5. **C** Venn diagrams showing the number of significantly DEGs in NC group vs. Model group and XKY-H group vs. Model group. **D** A cluster heatmap displays expression profiles of the significantly recovered DEGs by XKY-H treatment among the overlapped DEGs profiles in (**C**). **E** The top 20 of GO-biological process enrichment of the recovered DEGs profiles performed by DAVID online tool. **F** A cluster heatmap displays expression profiles of the recovered DEGs enriched in the cholesterol synthesis process. **G** Key DEGs (red text) involved in the process of cholesterol synthesis. (H) Relative mRNA expression of key DEGs involved in cholesterol synthesis process. Data were shown as means ± SD (*n* = 3, *P < 0.05, **P < 0.01, ***P < 0.001 vs. the Model group)

To explore the underlying mechanisms contributing to the improved hepatic glycolipid metabolism of XKY in *db/db* mice, we carried out transcriptomics analysis of liver tissues. The XKY-H group was selected as the representative XKY intervention group for transcriptomics analysis as well as further mechanistic investigation. In our study, a total of 5067 DEGs were identified in the Model group versus the NC group, 2603 of which were upregulated and 2464 of which were downregulated (Fig. 4A). Compared to Model group, 1154 DEGs were identified in the XKY-H group, 595 of which were upregulated and 559 of which were downregulated (Fig. 4B). The Venn diagram showed that there were 563 overlapping genes, among which a total of 418 remarkably up- or downregulated genes in the Model group were significantly recovered by XKY-H treatment (Fig. 4C). Figure 4D displays a heatmap of the DEGs that were reversed by XKY-H treatment which were considered potential therapeutic targets of XKY in this study. Using the DAVID online tool, transcriptomics-based enrichment analysis was further conducted to reveal the potential mechanisms of those genes. As shown in Fig. 4E, the biological process of GO enrichment analysis results revealed that the cholesterol biosynthetic process was significantly enriched. These results demonstrated that the beneficial effects of XKY on *db/db* mice may be associated with regulation of the cholesterol biosynthetic process. Then we conducted clustering analysis of the involved DEGs. Interestingly, ten DEGs encoding key enzymes involved in cholesterol synthesis were significantly upregulated in the Model group and downregulated upon XKY-H treatment (Fig. 4F and G). Next, we performed RT-qPCR to validate the expression of these genes, and the results demonstrated that the *Hmgcr*, *Mvk*, *Mvd*, *Idi1*, *Fdps*, *Sqle*, *Lss*, *Cyp51*, *Hsd17b1* and *Dhcr24* mRNA expression levels of were significantly increased in the Model group compared with the NC group, and were downregulated by XKY-H treatment compared with the Model group (Fig. 4H). These data suggest that XKY downregulates the cholesterol biosynthetic process to regulate metabolite homeostasis the in liver.

### XKY treatment altered the gut bacterial composition and improved the intestinal epithelial barrier damage in ***db/db*** mice

To address the potential involvement of the gut microbiota in mediating XKY improved metabolism in *db/db* mice, we performed sequencing of the 16 S rRNA V3-V4 region of feces from the NC, Model and XKY-H treatment groups. After removing the low‐quality sequences, we obtained 956,199 high‐quality sequences and 486 OTUs from 18 detected samples at a 97% homology cut‐off for subsequent analysis.

**Fig. 5:**
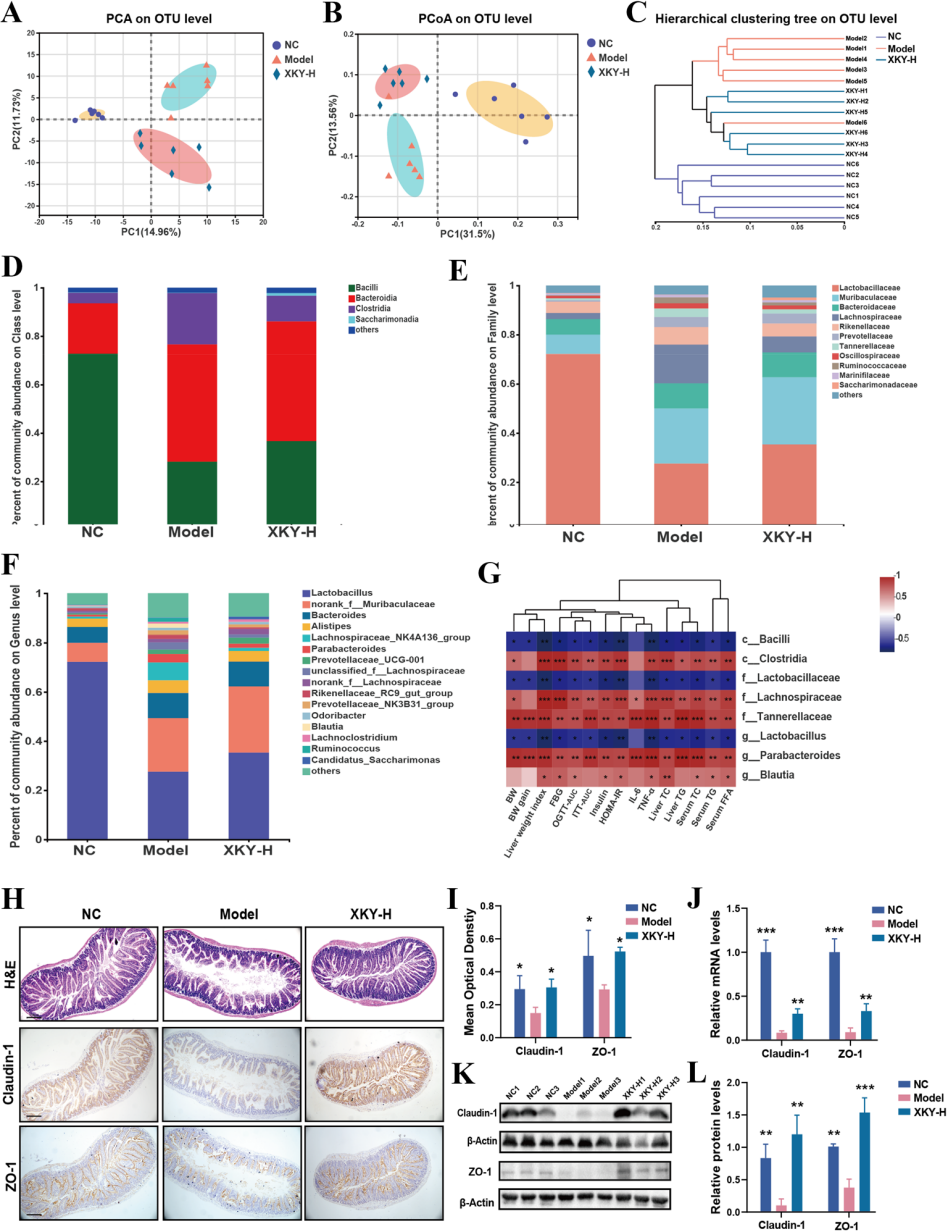
Effect of XKY treatment on gut microbiome composition and intestinal tissue in *db/db* mice. **A** PCA on OTU level of the NC, MOD and XKY-H groups. **B** PCoA of an unweighted UniFrac distance matrix on OTU level in the NC, Model and XKY-H groups. **C** Hierarchical clustering of an unweighted UniFrac distance matrix on OTU level in the NC, Model and XKY-H groups. Taxonomic composition of gut microbiome in the rats at the class (**D**), family (**E**), and genus (**F**) levels. **G** Pearson’s correlation analysis between the metabolic related parameters and the key differential gut microbiota. **H** and **I** H&E staining and Immunohistochemistry analysis of intestinal tissue specimens in the NC, MOD and XKY-H groups. Representative images were presented. Scale bars represent 100 μm. **J** The mRNA expression of Claudin-1 and ZO-1 of intestinal in NC, MOD and XKY-H groups detected by RT-qPCR (K) and **L** Western blotting analysis of Claudin-1 and ZO-1 of intestinal in NC, MOD and XKY-H groups. Data were shown as means ± SD (*n* = 3–6, *P < 0.05, **P < 0.01, ***P < 0.001 vs. the Model group)

The β-diversity, principal component analysis (PCA) and principal co‐ordinates analysis (PCoA) at the OTU level were conducted to analyze the gut microbiota compositional discrimination. The gut microbiota structure of the XKY-H group can be separated from that of the Model group (Fig. 5A and B). Additionally, hierarchical clustering analysis at the OTU level showed that the samples clustered closely with the treatment. Importantly, samples from the XKY-H treatment group differed from those of the Model group, and similar to the NC group (Fig. 5C). These results indicate that XKY administration altered the overall structure of the gut bacteria.

To investigate the XKY regulated bacterial taxa, we next compared the bacterial composition of each different group at the taxonomic level. We found that at the class level, the abundance of *Clostridia* was increased in the Model group and decreased in the XKY-H treatment group, whereas the XKY-H treatment group showed an increasing tendency of *Bacilli* which decreased in the Model group (Fig. 5D and Additional file 1: Fig. S1). In addition, at the family level, the abundance of *Lactobacillaceae* was significantly decreased in the Model group and showed an increasing tendency in the XKY-H treatment group (Fig. 5E and Additional file 1: Fig. S1). The abundance of *Lachnospircaeae* were markedly increased in the Model group and dramatically decreased upon XKY treatment, While the abundance of *Tannerellaceae* and *Ruminococcaceae* also showed a declining tendency in the XKY-H group with no statistic difference (Fig. 5E and Additional file 1: Fig. S1). Furthermore, as shown in Fig. 5F and Additional file 1: Fig. S1, at the genus level, *Lactobacillus* was significantly decreased in the Model group and showed an increasing tendency in the XKY treatment group. The relative abundance of the *Parabacteroides* and *Blautia* showed obvious elevation in the Model group and decline in the XKY-H treatment group (Fig. 5F and Additional file 1: Fig. S1). Although there was no statistic difference, the relative abundance of *Lachnospiraceae_NK4A136 group* and *unclassified_f_Lachnospiraceae* showed an increasing tendency in the Model group and a declining tendency in the XKY-H group (Fig. 5F and Additional file 1: Fig. S1).

To further probe into the key gut bacteria that potentially contribute to the beneficial effects of XKY, correlation analysis was performed to determine the potential association of bacterial abundance with metabolism-related phenomes. Notably, the relative abundance of *Bacilli* at class level, *Lactobacillaceae* at the family level and the promising probiotic *Lactobacillus* at the genus level which showed increasing tendency in the XKY-treated mice exhibited a definite inverse correlation with the values of FBG, OGTT-AUC, ITT-AUC, insulin, HOMA-IR, inflammatory factors (IL-6 and TNF-α), liver TC and TG and serum TC, TG and FFA (Fig. 5G). In addition, we observed that some other microbiota, including *Clostridia* at the class level, *Lachnospircaeae* and *Tannerellaceae* at the family level and *Parabacteroides* and *Blautia* at the genus which were depleted in XKY-treated mice, were positively correlated with the values of FBG, OGTT-AUC, ITT-AUC, insulin, HOMA-IR, inflammatory factor (IL-6 and TNF-α), liver TC and TG and serum TC, TG and FFA (Fig. 5G). These results further inferred that the involvement of the gut microbiota was closely related to metabolism-associated parameters, which might contribute to the therapeutic effects of XKY in *db/db* mice.

Gut dysbiosis can increase the intestinal barrier permeability, allowing for the influx of microbial metabolites across the epithelial layer and leading to crosstalk between the microbiota and distal target organs, which is commonly observed in metabolic diseases. Thus, we next assessed whether XKY had a beneficial effect on the intestinal barrier in *db/db* mice. First, we conducted HE staining to examine the effects of XKY on the small intestine and we observed an obviously abnormal villus structure of the small intestine epithelium including disorganized, collapsed and lower villus height and crypt depth in the Model group. These disorders were all restored after XKY-H treatment (Fig. 5H), suggesting that XKY ameliorated intestinal epithelial villus damage in the intestinal epithelium. Next, to further detect the effect of XKY on small intestine epithelial permeability, we carried out IHC, Western blotting, and RT-qPCR to measure the protein and mRNA expression levels of tight junction proteins. Accordingly, both the protein and mRNA expression levels of Claudin-1 and ZO-1 were diminished in the Model group, compared with the NC group, and markedly increased upon XKY treatment (Fig. 5H–L). Taken together, these results demonstrate that administration of XKY remodels the structure of the gut flora, alleviates gut dysbiosis and diminishes intestinal epithelial barrier damage in *db/db* mice.

### XKY promotes BA synthesis via LCA/DCA-FXR-FGF15 and regulates AA metabolism in *db/db* mice

**Fig. 6:**
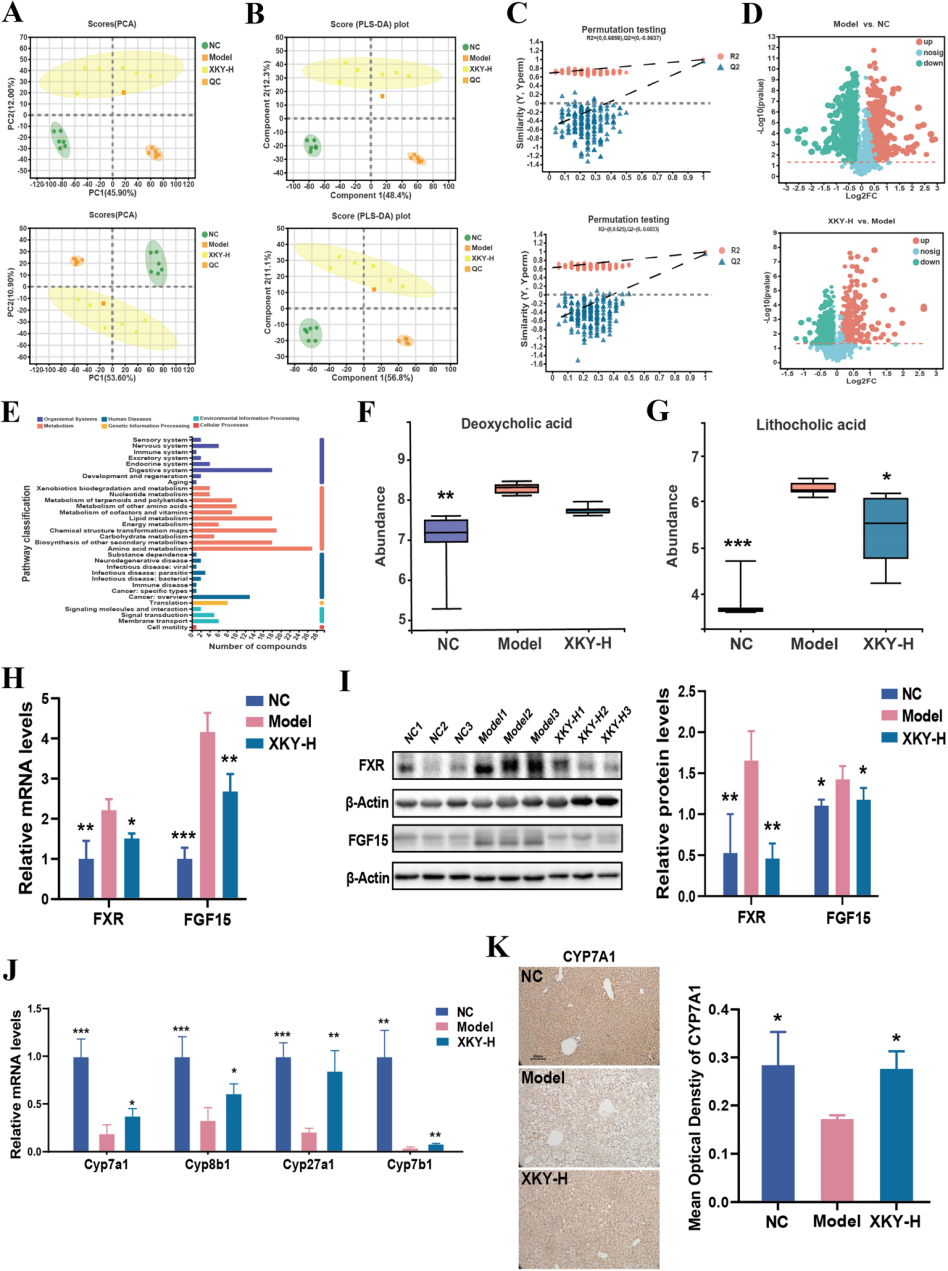
Effect of XKY treatment on fecal metabolomics in *db/db* mice. PCA (**A**) and OPLS-DA (**B**) of the NC, Model and XKY-H groups in ESI + mode (up) and ESI- mode (down). Each point represents one sample in the indicate groups, and the distance between points represents the similarity between the samples. **C** Model verification map of PLS-DA in ESI + mode (up) and ESI- mode (down). The ordinate represents the R2 and Q2 replacement test values, and the two dashes represent the regression lines of R2 and Q2 respectively. **D** Volcano plot of the differential metabolites of the NC group vs. Model group (up), XKY-H group vs. Model group (down). Each point in the figure represents a specific metabolite. Red color, green color, and blue color points indicated the significantly upregulated metabolites, downregulated metabolites and not significantly changed metabolite respectively. **E** Pathway classification of the differential metabolites. The relative abundance of lithocholic acid (**F**) and deoxycholic acid (**G**). **H** The mRNA expression of FXR and FGF15 of intestinal in NC, Model and XKY-H groups detected by RT-qPCR. **I** The protein expression of FXR and FGF15 of intestinal in NC, Model and XKY-H groups detected by Western blotting. **J** The mRNA expression of Cyp7a1, Cyp8b1, Cyp27a1 and Cyp7b1 of liver in NC, Model and XKY-H groups detected by RT‐qPCR. **K** Immunohistochemistry analysis of Cyp7a1 of liver in NC, Model and XKY-H groups. Scale bars represent 100 μm. Data were shown as means ± SD (*n* = 3–6, *P < 0.05, **P < 0.01, ***P < 0.001 vs. the Model group)

Since the intestinal microbiota interacts with the host via the complicated host-microbe-metabolic axis, we next conducted untargeted metabolomics on fecal samples from NC, Model and XKY-H group mice to reveal the potential mechanism that mediated the therapeutic effects of XKY. A principle component analysis (PCA) model was used to determine the principal component scores, as shown in Fig. 6A, there were clear group separations between the NC group and the Model group as well as that between the Model group and the XKY-H group in both electrospray ionization (ESI + and ESI−) modes. In addition, the orthogonal partial least squares discriminant analysis (PLS-DA) also showed significant distinctions among the NC, Model and XKY-H groups (Fig. 6B). The over-fitting in the PLS-DA model was controlled using seven-round cross validation and 200 repetitions of RPT based on the R2 and Q2 values. In ESI + mode, the R2 and Q2 values of the PLS-DA model in the comparison of the NC, Model and XKY-H groups were 0.6858 and − 0.5637, respectively (Fig. 6C). In ESI− mode the R2 and Q2 values in the PLS-DA model comparing the XKY-H and Model groups were 0.625 and − 0.6033, respectively (Fig. 6C). These results indicated that the PLS-DA models were consistent and reliable. These data suggested that XKY treatment induced a significant change in metabolic profile compared with the Model group.

Subsequently, metabolites with a VIP (variable importance in the projection) > 1 and p < 0.05 between NC and Model groups or between the XKY-H and Model groups were considered as differential metabolites. In total, 4061 (Model vs. NC) and 2830 (XKY-H vs. Model) differential spectra were identified in mouse intestines in ESI + mode, while 2247 (Model vs. NC) and 1789 (XKY-H vs. Model) were altered in ESI− mode. Then the differential metabolites between the Model and NC groups as well as between the XKY-H and Model group were visualized using volcano plots (Fig. 6D). These metabolites are mainly associated with metabolic pathways, including amino acid metabolism, lipid metabolism and energy metabolism. (Fig. 6E). Our 16 S rRNA sequence results revealed a downregulation of *Clostridia* and *Lachnospircaeae* upon XKY-H treatment, which were reported to be increased in mice fed a high-fat diet, related to secondary bile acid production and positively correlated with the levels of DCA and LCA, which acted as activators of FXR[26]. This evidence prompted us to compare the relative abundance of DCA and LCA in different groups. As expected, a significant accumulation of DCA and LCA in the Model group mice and a dramatic decrease in LCA in the XKY-H group were observed (Fig. 6F). Although not significant, DCA showed a declining tendency in the XKY-H treatment group (Fig. 6G). It was reported that DCA and LCA could activate FXR in small intestine and induce the expression of FGF15 to inhibit hepatic BA synthetic enzymes[27]. We then detected the expression levels of FXR and FGF15 in the small intestine and found that both the mRNA and protein expression of FXR and FGF15 were increased in the Model group and decreased in the XKY-H treatment group (Fig. 6H and I). Moreover, the mRNA expression of hepatic *Cyp7a1*, *Cyp8b1*, *Cyp27a1* and *Cyp7b1* was decreased in the Model group and increased in the XKY treatment group (Fig. 6J). Additionally, we also selected the BA synthetic key enzyme CYP7A1 to detect the protein expression by IHC assay and found that it was increased in the Model group and decreased in the XKY-H group (Fig. 6K). These results demonstrated that XKY could reverse the elevated fecal DCA and LCA induced intestinal FXR-FGF15 signalling to promote hepatic BA synthesis.

**Fig. 7:**
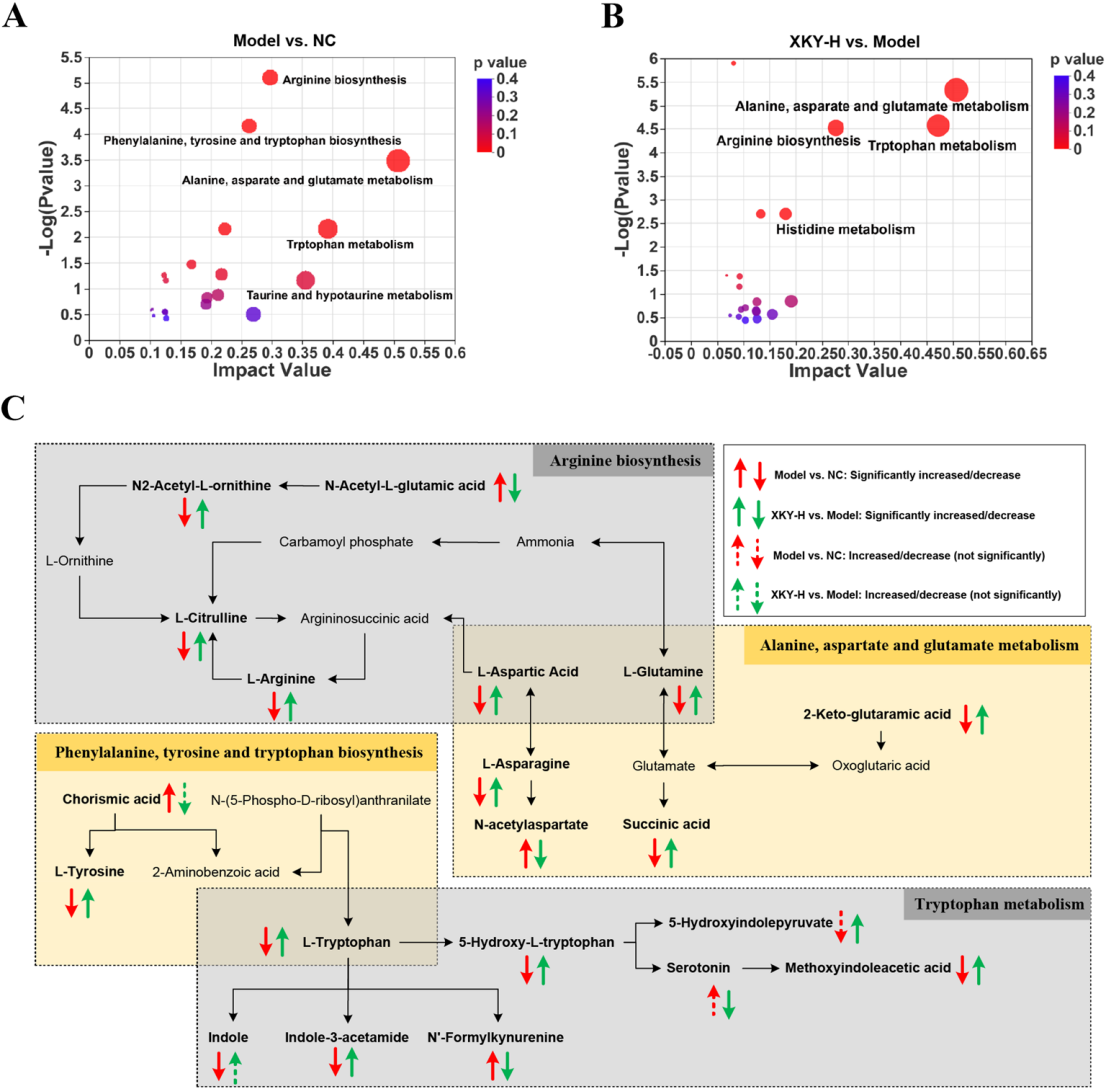
Analysis of pathway related to fecal differential metabolites. KEGG topology analysis on the differential metabolites of the NC group vs. Model group (**A**), XKY-H group vs. Model group (**B**). **C** Schematic illustration of arginine metabolism, alanine, aspartate and glutamate metabolism, phenylalanine, tyrosine and tryptophan biosynthesis, and tryptophan metabolism by fecal untargeted metabolomics from NC, Model and XKY-H treatment groups. These four pathways are affected in both Model vs. NC and XKY-H treatment vs. Model groups. These metabolites are closely related to each other and the previous literatures reported relationships between each metabolite were showed in black arrows

Next, to further explore the function of the differential metabolites, we performed topology analysis on differential metabolites between the Model and NC groups or XKY-H and Model groups. As shown in Fig. 7A and B, arginine biosynthesis, alanine, aspartate and glutamate metabolism, phenylalanine, tyrosine and tryptophan biosynthesis, and tryptophan metabolism were significantly enriched, indicating that those amino acid metabolism processes were tightly associated with the pathological conditions of *db/db* mice as well as respond to XKY-H treatment. Based on our results, literature and database information, the relationship and the overall changes in the differential metabolites involved in those significantly changed metabolic pathways are visualized in Fig. 7C. In particular, the levels of L-Glutamine and L-Aspartic Acid were decreased in the Model group and increased in the XKY-H treatment group, which were the overlapped differential metabolites in the arginine biosynthesis pathway and the alanine, aspartate and glutamate metabolism pathways (Additional file 1: Fig. S2A and B).

In addition, for the arginine biosynthesis pathway, L-Arginine, N2-Acetyl-L-ornithine and L-Citrulline levels were significantly decreased in the Model group and dramatically upregulated in the XKY-H treatment group (Additional file 1: Fig. S2C-E). The N-Acetyl-L-glutamic acid level was significantly increased in the Model group and decreased in the XKY-H treatment group (Additional file 1: Fig. S2F). In the alanine, aspartate and glutamate metabolism pathways, 2-Keto-glutaramic acid, L-Asparagine, and succinic acid were decreased in the Model group and increased in the XKY-H treatment group (Additional file 1: Fig. S2G–I). The N-acetylaspartate level was increased in the Model group and decreased in the XKY-H treatment group (Additional file 1: Fig. S2J). In the phenylalanine, tyrosine and tryptophan biosynthesis pathway, L-Tryptophan and L-Tyrosine levels were decreased in the Model group and increased in the XKY-H treatment group (Additional file 1: Fig. S2K and L), while the chorismic acid level was significantly increased in the Model group and showed a declining tendency in the XKY-H treatment group (Additional file 1: Fig. S2M). In the tryptophan metabolism pathway, in addition to that of L-Tryptophan, methoxyindoleacetic acid, indole-3-acetamide and 5-Hydroxy-L-tryptophan level were significantly decreased in the Model group and increased in the XKY-H treatment group (Additional file 1: Fig. S2N–P), while the N′-Formylkynurenine level was increased in the Model group and decreased in the XKY-H treatment group (Additional file 1: Fig. S2Q). Additionally, the indole level was notably decreased in the Model group and partly elevated after XKY-H ingestion (Additional file 1: Fig. S2R). Serotonin and 5-Hydroxyindolepyruvate levels showed an increasing/decreasing tendency in the Model group and were obviously reversed upon XKY-H treatment (Additional file 1: Fig. S2S and T). Taking this evidence together, we hypothesize that XKY affects the gut microbiota structure and substantially inhibits DCA/LCA-FXR-FGF15 signaling to promote BA synthesis and regulate AA metabolism to alleviate metabolic features in *db/db* mice.

### Correlations among key intestinal microbes, metabolites and metabolism-related parameters

**Fig. 8:**
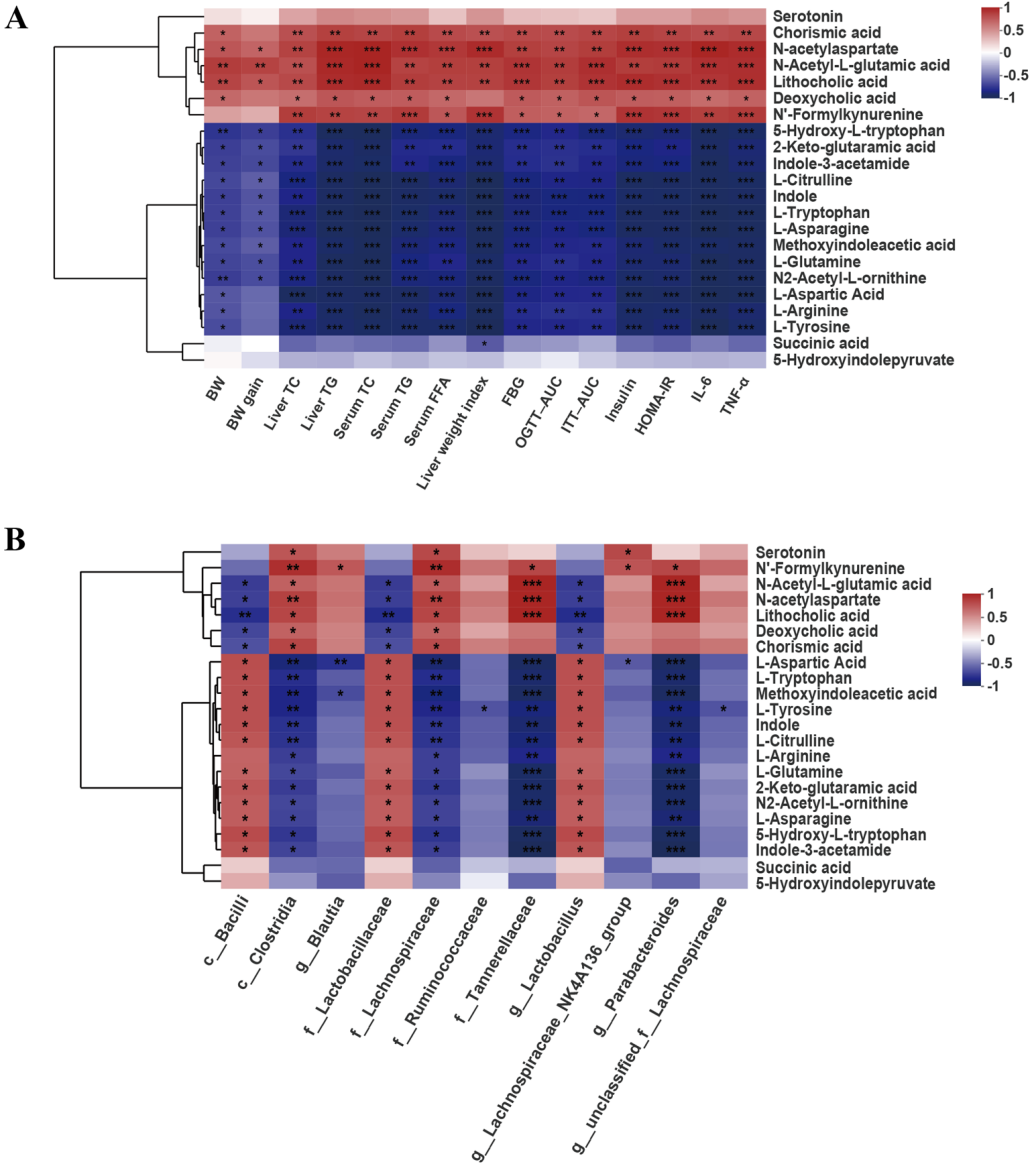
Correlations among differential gut Microbiota, key differential fecal metabolites and metabolic related parameters. **A** Correlation between metabolic related parameters and the key differential fecal metabolites. **B** Correlation between the relative abundances of differential gut microbiota and the key differential fecal metabolites. Different colors represent correlation level, *n* = 4–6, *P < 0.05, **P < 0.01, ***P < 0.001

To further elucidate the relationship between of the differential metabolites and the metabolism-related parameters, Pearson correlation analysis was performed. The concentrations of DCA, LCA, chorismic acid, N-acetylaspartate, N-Acetyl-L-glutamic acid and N-Formylkynurenine were positively correlated with liver TC and TG, serum TC, TG and FFA, FBG, OGTT-AUC, ITT-AUC, insulin, HOMA-IR and pro-inflammatory factors (TNF-α and IL-6) levels (Fig. 8A). The concentrations of 5-Hydroxy-L-tryptophan, 2-Keto-glutaramic acid, indole-3-acetamide, L-Citrulline, indole, L-Tryptophan, L-Asparagine, methoxyindoleacetic acid, L-Glutamine, N2-Acetyl-L-ornithine, L-Aspartic Acid, L-Arginine and L-Tyrosine were negatively correlated with liver TC and TG, serum TC, TG and FFA, FBG, OGTT-AUC, ITT-AUC, insulin, HOMA-IR and proinflammatory factor (TNF-α and IL-6) levels (Fig. 8A).

**Fig. 9:**
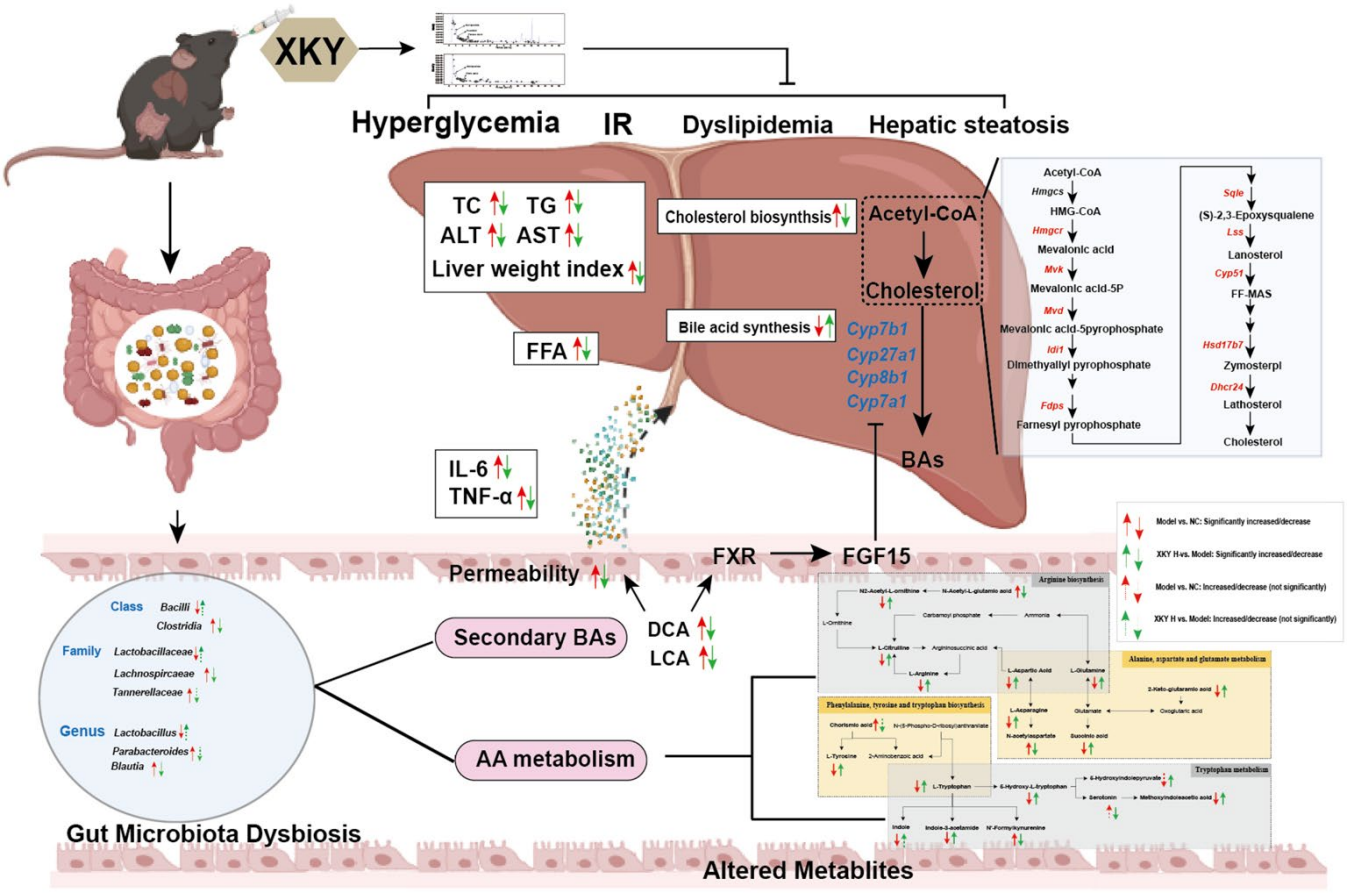
Model depicts the improvement of metabolic features in *db/db* mice upon XKY treatment. XKY mitigates hyperglycemia, IR, hyperlipidemia, inflammation and hepatic steatosis. Mechanistically, XKY decreases the hepatic cholesterol synthesis by inhibiting *Hmgcr*, *Mvk*, *Mvd*, *Idi1*, *Fdps*, *Sqle*, *Lss*, *Cyp51*, *Hsd17b1* and *Dhcr24* gene expression. In addition, XKY decreases secondary bile acid producing bacteria (*Clostridia* and *Lachnospircaeae*) to mainly result in decreasing the levels of proinflammatory factors (IL-6 and TNF-α) and secondary bile acids (lithocholic acid (LCA) and deoxycholic acid (DCA), and maintaining intestinal epithelial homeostasis, and to further promote hepatic bile acid synthesis by inhibiting the LCA/DCA-FXR-FGF15 signaling pathway followed by down-regulating *Cyp7a1*, *Cyp8b1*, *Cyp27a1* and *Cyp7b1* gene expression. Furthermore, XKY regulates amino acid metabolism including arginine biosynthesis, alanine, aspartate and glutamate metabolism, phenylalanine, tyrosine and tryptophan biosynthesis, and tryptophan metabolism likely by increasing *Bacilli*, *Lactobacillaceae* and *Lactobacillus*, and decreasing *Clostridia*, *Lachnospircaeae*, *Tannerellaceae* and *Parabacteroides* abundances. This figure was created with BioRender.com.

The correlations between the differential intestinal microbiota and the differential metabolites were also analyzed. As shown in Fig. 8B, the predominant microbiota *Bcilli*, *Lactobacillaceae* and *Lactobacillus* in the XKY group displayed positive correlations with L-Aspartic Acid, L-Tryptophan, methoxyindoleacetic acid, L-Tyrosine, Indole, L-Citrulline, L-Glutamine, 2-Keto-glutaramic acid, N2-Acetyl-L-orninthine, L-Asparagine, 5-Hydroxy-L-tryptophan and indole-3-acetamide and negative correlations with DCA, LCA, chorismic acid and N-acetylaspartate (Fig. 8B). The dominant genera in the Model group, including *Clostridia*, *Lachnospiraceae*, *Tannerellaceae* and *Parabacteroides* exhibited significant correlations with more than three differential metabolites (Fig. 8B). The results of the above correlation analysis revealed a close relationship among the gut microbiota, metabolites, and metabolism-related parameters. Collectively, these correlations provide evidence that the enrichment of *Bacilli*, *Lactobacillaceae* and *Lactobacillus*, and the downregulation of *Clostridia*, *Lachnospiraceae*, *Tannerellaceae* and *Parabacteroides* accompanied by declines in DCA and LCA. Improvements in arginine biosynthesis, alanine, aspartate and glutamate metabolism, phenylalanine, tyrosine and tryptophan biosynthesis, and tryptophan metabolism have important roles in XKY ameliorating metabolic have important roles in XKY ameliorating metabolic disorders (Fig. 9).

## Discussion

Accumulating clinical and experimental evidence indicates that TCM plays an effective role the in the prevention and treatment of metabolic diseases, including diabetes, obesity, hyperlipidemia and NAFLD. In the present study, we investigated the effect of the TCM formula XKY on metabolic disorders in *db/db* mice, an efficient genetic model of metabolic diseases that mimics histologic as well as metabolic features including IR, disrupted glucolipid metabolism and excessive lipid accumulation [28]. Our results first demonstrated that XKY treatment alleviated hyperglycemia and IR in *db/db* mice. Hepatic histological and biochemical experimental results confirmed that XKY intake induced liver injury and decreased lipid accumulation, whereas it increased glycogen content. Moreover, XKY treatment alleviated liver function, lipid profile and inflammation. Consistent with the definite hypolipidemic effects suggested by the mouse phenotype, hepatic transcriptome results revealed a significant downregulation of the cholesterol biosynthetic process in response to XKY treatment. Our RT-qPCR results validated that XKY treatment decreased the hepatic expression of *Hmgcr*, *Mvk*, *Mvd*, *Fdps*, *Sqle*, *Lss*, *Cyp51*, *Hsd17b1*, and *Cyp39a1*, which are involved in the cholesterol biosynthetic process. By carrying out a 16 S rRNA sequencing analysis, we found that XKY treatment restored the disordered gut microbiota, increased the relative abundance of beneficial microbiota *Lactobacillus*, *Bacilli* and *Lactobacillaceae*, decreased the relative abundance of *Clostridia*, *Lachnospircaeae*, *Tannerellaceae*, *Parabacteroides* and *Blautia*, and improved the intestinal structure. Moreover, fecal untargeted metabolomic analysis revealed that XKY treatment decreased DCA and LCA levels to promote hepatic BA synthesis by inhibiting the intestinal FXR-FGF15 signaling pathway, and regulated AA metabolism including arginine biosynthesis, alanine, aspartate and glutamate metabolism, phenylalanine, tyrosine and tryptophan biosynthesis, and tryptophan metabolism in *db/db* mice. Notably, correlation analysis showed an obvious association of the metabolic related features, intestinal flora and key metabolites, indicating a critical role of gut microbiota in XKY treatment.

A substantial body of literature has described the therapeutic effects of “medicine food homology” herbs on metabolic diseases. For example, *Polygonatum sibiricum* is a well-known traditional medicinal herb and functional food in China [29], and its active constituent *Polygonatum sibiricum* polysaccharides (PSP), have been proven to decrease the fasting blood glucose (FBG) and glycated hemoglobin levels in a dose dependent manner, and improve the clinical symptoms such as polydipsia, polyuria in streptozotocin-induced diabetes mellitus rats [30], and prevent high-fat diet (HFD) and streptozotocin-injection induced T2DM by regulating the gut microbiota [31]. *Gardenia jasminoides* Ellis was originally recorded in Shennong’s Classic of Materia Medica more than 2000 years ago and it is the main component of many classic TCM formulas, such as Huanglian Jiedu decoction. *Gardenia jasminoides* Ellis and its main chemical ingredients have been widely used to treat inflammation [32] and various metabolic diseases, such as diabetes [33], hyperlipidemia, hepatic lipid accumulation [34] and NAFLD [35]. *Angelica sinensis (Oliv.) Diels.* is a well-known TCM, and also a health food for women’s care, as well as a dietary supplement [36]. A previous study reported that *Angelica sinensis* polysaccharide (ASP) isolated from *Angelica sinensis (Oliv.) Diels*, decreased hyperglycemia, stimulated insulin secretion, promoted hepatic glycogen synthesis and attenuated fat accumulation, indicating that ASP could be used as the functional food or in prescriptions for the prevention or treatment of liver diseases and diabetes [37]. *Crataegus pinnatifida Bunge* was reported to decrease blood glucose, TC and TG levels to protect against STZ-induced T2DM [38]. XKY consists of nine “drug homologous food” herbs that are widely used in the treatment of metabolic diseases and it shows a potential protective role in metabolic disorder patients in our clinical trial. However, no literature has been published on the effects of XKY. This is the first study to evaluate the efficacy of XKY on glucolipid metabolism and explore the underlying mechanism in *db/db* mice. Our results demonstrated that XKY effectively decreased FBG, liver index, OGTT-_AUC_, ITT-_AUC_ and insulin levels and improved IR. In addition, our results showed that XKY treatment significantly diminished hepatic lipid deposition and steatosis accompanied by improved liver function (ATL and AST), lipid profile (TC and TG) and inflammation (TNF-α and IL-6) in *db/db* mice. The active ingredients of the individual herbs in XKY were reported to possess a wide variety of bioactivities, such as antidiabetic, anti-inflammatory, cholesterol-lowering and liver injury-protective activities. According to the Chinese Pharmacopoeia, geniposide, puerarin, ferulic acid, citric acid and rutin are the main active compounds of the individual herbs in XKY, and most of these main active ingredients were indeed also detected in XKY in this study. Our results are consistent with some previous studies. In particular, geniposide is the main active compound of *Gardenia jasminoides* Ellis and it was reported to decrease FBG and regulate dysfunctions in phenylalanine metabolism, tryptophan metabolism and secondary bile acid biosynthesis pathways in feces of a T2DM rat model [33]. The main active ingredient of *Pueraria lobata (Willd.) Ohwi* is puerarin, and recent evidence indicates that puerarin ameliorates glucose and lipid metabolism dysfunction in hepatic cells [39] and suppresses the hepatic gluconeogenesis via activation of the PI3K/Akt signaling pathway in diabetic rats [40]. *Polygonati Odorati Rhizoma*, is used both for food and medicine to prevent and treat metabolic disorders, and its extraction was verified to reduce FBG and TG levels and improve glucose tolerance and insulin tolerance to protect C57BL/6 mice from diet-induced metabolic disorders [41]. Ferulic acid and polysaccharides are the main bioactive constituents of *Angelica sinensis (Oliv.) Diels*, and ferulic acid is a marker content used to determine the quality of *Angelica sinensis (Oliv.) Diels* according to the Chinese Pharmacopoeia [42]. In a recent study, Qi et al. investigated the effect of ferulic acid on STZ-induced diabetic nephropathy rats and found that ferulic acid improved inflammation and oxidative stress, reduced FBG, TC and TG, and attenuated podocyte injury [43]. Thus, we speculate that these active ingredients are responsible for the therapeutic action of XKY. Taken together, our data indicate that XKY is a reliable treatment strategy that effectively downregulates FBG levels, improves IR, reduces liver lipid accumulation, decreases both hepatic and serum lipid contents and ameliorates inflammation in *db/db* mice.

The liver is a crucial organ for the maintenance of normal glucose and lipid metabolism and ectopic hepatic lipid deposition leads to IR, which is an important predictive factor of metabolic diseases such as diabetes and NAFLD [44–46]. Cholesterol metabolism disorders has been reported to be closely related to hepatic lipid deposition. Cholesterol biosynthesis is under tight regulation mainly in the liver starting from acetyl-CoA through approximately 30 steps that comprise more than 20 enzymes, such as HMG-CoA reductase Hmgcr, Mvk, Mvd, Fdps and Sqle [47]. Despite the protective effects of XKY on hepatic glucolipid metabolism, the underlying molecular mechanisms remain unknown. In this study, we performed a hepatic transcriptome analysis and demonstrated that XKY significantly changed the overall transcriptome expression profile. Consistent with the dramatically decreased levels of TC and TG, XKY treatment significantly downregulated cholesterol biosynthetic process. Moreover, our RT-qPCR results verified that *Hmgcr*, *Mvk*, *Mvd*, *Idi1*, *Fdps*, *Sqle*, Lss, *Cyp51*, *Hsd17b1* and *Dhcr24*, which are involved in the cholesterol biosynthetic process were increased in Model group as compared with the NC group, and those genes were decreased by XKY treatment compared with the Model group. Our results are in accordance with some previous studies. It was reported that Qing Gan San (QGS) formula comprises which also consisting of *Polygonatum sibiricum* and the leaves of Morus alba L could significantly reduce the levels of total cholesterol and triglycerides in both serum and liver tissue by suppressing the transfer of Srebp-1 to the nucleus from the cytoplasm to inhibit lipogenesis [48]. *Crataegus pinnatifida* Bunge treatment can lower serum TC and TG levels in a hypercholesterolemic rabbit model [49]. Study has proven that the cholesterol-lowering effect of *Crataegus pinnatifida* Bunge may be due to up-regulation of CYP7A1 or its regulation of bile acid synthesis in the hypercholesterolemic rat model [50]. In addition, the daily food intake data demonstrated that XKY did not change energy acquisition. Collectively, it can be deduced that XKY regulates hepatic lipid homeostasis to ameliorate hepatic steatosis by downregulating cholesterol biosynthetic process-related genes in the livers of *db/db* mice.

Accumulating studies have revealed a correlation between dysbiosis and metabolic diseases such as IR, obesity, diabetes, and NAFLD [51]. We therefore explored the potential involvement of the gut microbiota in mediating XKY improved glucolipid metabolism in *db/db* mice. As expected, XKY treatment affected the overall structure of the gut microbiota, and the β-diversities in the XKY treatment group showed a greater difference from those in the Model group. *Lactobacillus* are widely considered promising probiotics, and the *Lactobacillus* genus are members in *Lactobacillaceae* family and *Bacilli* class. *Lactobacillus* represents lower abundance in diabetic and obese rats and greater abundance in diabetic rats that were under treatment [52]. In addition, lower abundance *Lactobacillus* was also observed in people with NAFLD, and ingestion of *Lactobacillus* was regarded as a useful therapeutic option for the treatment of NAFLD to reduce lipid levels (TC and TG) and inflammatory cytokines (TNF-α, IL-1β and IL-6) [10, 53]. Consistent with these observations, we noted that the relative abundances of *Bacilli* at the class level, *Lactobacillaceae* at the family level and *Lactobacillus* at the genus level were decreased in the Model group and showed increasing tendency in the XKY-treated mice. Importantly, the gut microbiota exhibited a definite inverse correlation with FBG, OGTT-AUC, ITT-AUC, insulin, HOMA-IR, inflammatory factors (IL-6 and TNF-α), liver TC and TG and serum TC, TG and FFA. This result is also in accordance with the results of previous studies, which suggest that the main active substances in *Polygonatum sibiricum* has an excellent hypoglycemic and Hypolipidemic effects and could promote the growth of beneficial bacteria such as *Lactobacillus *[54, 55]. In contrast, some other differential microbiota including *Clostridia* at the class level, *Lachnospircaeae* and *Tannerellaceae* at the family level and *Parabacteroides* and *Blautia* at the genus were elevated in the Model group and depleted upon XKY administration. A positive correlation between the gut microbiota and FBG; OGTT-AUC; ITT-AUC; insulin; HOMA-IR; inflammatory factor (IL-6 and TNF-α); liver TC and TG; and serum TC, TG and FFA levels was observed. Consistently, in a prior randomized cross-over trail, researchers found that inulin supplementation improved insulin sensitivity and decreased *Clostridia* in adults with overweight and obesity [56]. Wang et al. reported that HFD led to an accumulation of *Ruminococcaceae* and *Lachnospiraceae* and inhibition of *Lactobacillaceae*, and those results could be reversed accompanied by a remarkable reduction in plasma lipid levels by chlorogenic acid administration [57]. More interestingly, it was reported that many *Clostridia* taxa and *Lachnospiraceae* taxa are secondary BA-producing bacteria that possess the ability to regulate secondary BA biotransformation [26]. Importantly, the relative abundances of *Clostridia* and *Lachnospircaeae* were also found to be increased in mice in a high-fat diet-induced NAFLD mouse model and/or NAFLD patients [26, 58] and positively correlated with the levels of DCA and LCA [26]. These results suggested that XKY might decrease secondary BA-producing bacteria to regulate secondary BA biotransformation. *Parabacteroides* was increased in obese patients compared to lean subjects [59]. Several studies have demonstrated that the genus *Blautia* is a harmful microbe and is increased in NAFLD patients and high-fat diet (HFD)-fed obesity/NAFLD mice, and *Blautia* is positively correlated with NAFLD-related parameters such as BW gain, hepatic TC, hepatic TC, serum AST and serum AST [17, 58, 60]. Collectively, our current findings, in concert with previous results, indicated that the ameliorative effects of XKY on metabolic disorders might be, at least in part, by restoring the dysbacteriosis by upregulating the abundance of *Bacilli* at the class level, *Lactobacillaceae* at the family level and *Lactobacillus* at the genus level and downregulating the relative abundance of *Clostridia* at the class level, *Lachnospircaeae* and *Tannerellaceae* at the family level and *Parabacteroides* and *Blautia* at the genus level. Our results provide new insights into the potential roles of gut microbiota in mediating XKY-improved glucolipid metabolism in *db/db* mice.

Emerging evidence has highlighted the role of gut microbiota-associated metabolites in maintaining the homeostasis of host physiological function. In particular, gut microbes produce enzymes to regulate secondary bile acid metabolism, and the elevated fecal lithocholic acid (LCA) and deoxycholic acid (DCA) levels were reported to potentially destroy the gut barrier and increase the expression levels of inflammatory factors (TNF-α and IL-6), contributing to the development of NAFLD [61]. In addition, AAs such as tryptophan, phenylalanine and tyrosine and their metabolites from bacterial metabolism are involved in the pathogenesis of metabolic diseases [14]. For example, tryptophan metabolism was reported to regulate lipogenesis, low-grade chronic inflammation and energy expenditure contributing to metabolic improvement [62, 63]. Metabolic diseases showed a dysbacteriosis, thereby decreasing their abilities to metabolize tryptophan into its derivatives such as indole, which was found to prevent LPS induced alterations of cholesterol metabolism and alleviate liver inflammation in mice [64]. We therefore attempted to unravel the fecal metabolites that potentially mediate XKY-improved glucolipid metabolism in *db/db* mice. The PCA and PLS-DA of fecal untargeted metabolomics showed different metabolic profiles of the MOD and XKY treatment groups, indicating that XKY could modify the metabolic profiles in *db/db* mice. BAs act as important regulators of lipid and glucose metabolism, and inflammation [65]. Primary bile acids are synthesized in hepatocytes via the classical and alternative pathways. Then the primary bile acids are conjugated to form conjugated bile acid, followed by metabolism into secondary bile acids. Primary bile acids can be metabolized into secondary bile acids (DCA and LCA) by secondary BA-producing gut microbiota [65, 66]. DCA and LCA are potent endogenous ligands of FXR that can activate FXR in the small intestine and induce the expression of FGF15 to inhibit hepatic BA synthetic enzymes [27]. Consistently, we found that XKY treatment lowered the abundance of secondary BAs (DCA and LCA) and decreased the abundance of secondary BAs producing bacteria (*Clostridia* and *Lachnospiraceae*). Importantly, XKY treatment inhibited the mRNA and protein expression of FXR and FGF15 in the small intestine and elevated the expression of hepatic *Cyp7a1*, *Cyp8b1*, *Cyp27a1* and *Cyp7b1* mRNA. In addition, DCA and LCA were reported to potentially increase gut permeability and increase the exposure of the liver to gut-derived toxins, which can increase proinflammatory cytokines (TNF-α and IL-6), thereby promoting inflammation and hepatic steatosis in animal models of NAFLD [26, 66]. Moreover, our correlation analysis showed a positive correlation between LCA, DCA and *Clostridia*, *Lachnospiraceae*, proinflammatory factors (TNF-α and IL-6) as well as glucolipid related index. In previous studies, high DCA concentrations were observed in T2DM and NAFLD patients [65, 67]. Taken together, these results provided evidence that on the one hand, the decreased abundance of *Clostridia* and *Lachnospiraceae* in the feces upon XKY treatment may account for the lower fecal concentration of secondary BAs (DCA and LCA), increasing intestinal permeability (Caludin-1 and ZO-1) and decreasing proinflammatory factors. On the other hand, accumulation of fecal DCA and LCA may directly activate intestinal FXR-FGF15 signaling to inhibit hepatic BA synthesis, which could be mitigated by XKY treatment.

Intriguingly, our topology enrichment analysis on differential metabolites between both the Model group vs. NC group and XKY-H group vs. Model group demonstrated that arginine biosynthesis, alanine, aspartate and glutamate metabolism, phenylalanine, tyrosine and tryptophan biosynthesis, and tryptophan metabolism were significantly enriched. This indicated to us that those amino acid metabolism processes were tightly associated with the metabolic disorders as well as the respond to XKY treatment in *db/db* mice. In particular, in terms of arginine biosynthesis and alanine, aspartate and glutamate metabolism, L-Arginine, L-Glutamine, L-Citrulline, L-Aspartic Acid, L-Asparagine, succinic acid, N2-Acetyl-L-ornithine and 2-Keto-glutaramic acid levels were decreased in the Model group and reversed in the XKY-H treatment group, whereas N-Acetyl-L-glutamic acid (N-Acetyl-L-glutamate) and N-acetylaspartate were increased in the Model group and decreased in the XKY-H treatment group. Our current results are consistent with the following studies. L-Arginine, a nutritionally functional nonessential amino acid, was found to have the potential to prevent T2DM and enhance insulin sensitivity which might occur by regulating the L-arginine-nitric oxide pathway [68]. L-Glutamine, a major precursor for citrulline synthesis [69], could improve intestinal barrier function [70] and protect mice from non-alcoholic steatohepatitis in western-style diet-fed mice [71]. N2-Acetyl-L-ornithine, can be used to synthesize ornithine [72]. Ornithine and arginine are the main precursors of L-Citrulline, which is also an immediate precursor for the endogenous synthesis of arginine [73]. A previous study documented that citrulline supplementation prevented hypertriglyceridemia and attenuated liver fat accumulation in fructose-induced NAFLD [74]. L-aspartic acid was negatively correlated with hepatic TG level, and L-aspartic acid treatment facilitated the LKB1-AMPK axis, accelerated lipid and cholesterol absorption and inhibited oxidative stress in hepatocytes and intestinal cells, thus ameliorating NAFLD in obese mice [8]. L-Asparagine, is a glucogenic amino acid that can be converted to L-aspartic acid. To date, other studies have shown that serum asparagine was significantly lower in obese individuals and that reduced asparagine was associated with T2DM [75].

Phenylalanine, tyrosine and tryptophan are aromatic amino acids (AAAs) that can be metabolized into multiple aromatic acids by gut microbiota, and the imbalance of those AAAs was confirmed to be a contributing factor to metabolic diseases such as T2DM, NAFLD and obesity [76]. Chorismic acid, also known as chorismate, is a precursor and starting metabolite of branched pathway for the biosynthesis of AAAs, and a reduction in chorismate leads to lower production of serotonin (5-hydroxytryptamine, 5-HT) thereby playing an anti-obesity role [77, 78]. Consistently, our results showed that the chorismic acid level was increased in the Model group and showed a decreasing tendency upon XKY treatment.

L-Tryptophan, an essential aromatic amino acid, was reported to be reduced in obese subjects compared to non-obese controls [79]. Tryptophan supplementation could improve the intestinal barrier and attenuate liver steatosis in a diet-induced experimental NAFLD mouse model [80]. Consistently, we found that tryptophan was decreased in the Model group and increased in mice receiving XKY. Of note, the tryptophan metabolism pathway was also among the top enriched pathways. Tryptophan metabolism including kynurenine, 5-hydroxytryptamine (serotonin, 5-HT), and indole derivatives pathways, is closely related to the intestinal microbiota [81]. In low-grade inflammatory situations, overactivation of kynurenine pathway is considered to be involved in the onset of IR [63]. We found that the N′-Formylkynurenine level was increased in the Model group and decreased in the XKY treatment group, indicating a downregulated kynurenine pathway in response to XKY administration. In addition, the serum level of 5-HT was elevated in diabetic patients, and the increasing concentration in serum was considered as a marker of diabetic complications [82]. In this study, serotonin showed an increased tendency in the Model group and XKY significantly decreased the level of serotonin. However, 5-Hydroxy-L-tryptophan (5-HTP) (a direct precursor of 5-HT) and 5-Hydroxyindolepyruvate levels were decreased in the Model group and increased in the XKY treatment group, indicating that XKY treatment had an obvious effect on serotonin metabolism in *db/db* mice. In addition, tryptophan could also be directly metabolized into indole and its derivatives, which were reported to regulate hepatic lipogenesis, inflammation, glycolysis and intestinal barrier integrity [63]. A recent study identified that *Lactobacillus*-derived methoxyindoleacetic acid (5-Methoxyindoleacetate, 5-MIAA) can activate hepatic Nrf2 and promote the expression of genes involved in the cellular antioxidant ability to protect against liver injury [83]. In the present study, in the feces of the Model group mice, indole level and indole derivatives methoxyindoleacetic acid and indole-3-acetamide (IAM) were significantly decreased, and those results could be partly reversed by XKY treatment.

Moreover, our Pearson’s correlation analysis showed that most of the upregulated metabolites involved in AA metabolism upon XKY treatment were positively correlated with the abundance of *Bacilli*, *Lactobacillaceae* and *Lactobacillus* and negatively correlated with the abundance of *Clostridia*, *Lachnospircaeae*, *Tannerellaceae* and *Parabacteroides.* Likewise, we observed that the downregulated metabolites upon XKY treatment were negatively correlated with *Bacilli*, *Lactobacillaceae* and *Lactobacillus*, and positively correlated with *Clostridia*, *Lachnospircaeae*, *Tannerellaceae* and *Parabacteroides.* Taking this evidence together, the modulatory effects of XKY on AA metabolism might be exerted by increasing the abundances of *Bacilli*, *Lactobacillaceae* and *Lactobacillus*, and decreasing the abundances of *Clostridia*, *Lachnospircaeae*, *Tannerellaceae* and *Parabacteroides.*

There are a few limitations that should be considered in our results. We performed a UPLC-QE-MS assay to analysis the chemical profile of XKY and we will do quantitative analysis of the main components in our future research. We detected the relative abundance of the fecal metabolites using an untargeted metabolomics approach and we will further quantify the absolute levels of the promising metabolites using targeted metabonomic assays. Although the metabolites may permeate through the intestinal barrier, fecal metabolomes may not fully reflect the metabolites in serum and we will further detect the serum metabolomes. XKY treatment group showed an increasing/decreasing tendency of some intestinal bacteria which were decreased/increased in the Model group compared with that of the NC group. The role of these intestinal bacteria needs to be further confirmed by expanding the samples’ quantity in our future research. We found that XKY modulated gut microbiota dysbiosis and its metabolites in an in vivo mouse model, and we will further study its effects on the gut microbiota of metabolism in metabolic disease patients. Bile acid metabolism plays an important role in cholesterol homeostasis under complex regulation. In this study, we determined that XKY treatment decreased fecal LCA and DCA levels. The detailed influence of XKY on hepatic and fecal BA pools and the related regulatory mechanism need to be further investigated in our future study.

## Conclusions

In conclusion, our study provides experimental evidence to indicate that XKY elicits therapeutic effects on glucolipid metabolism in *db/db* mice. Mechanistically, XKY decreases the hepatic cholesterol biosynthetic gene expression. Importantly, this study validates that XKY alters the gut microbiota, reduces LCA and DCA levels to promote hepatic BA synthesis by inhibiting the FXR-FGF15 signaling pathway; and regulates AA metabolism (arginine biosynthesis; alanine, aspartate and glutamate metabolism; phenylalanine, tyrosine and tryptophan biosynthesis; and tryptophan metabolism), which play an important role in the pathological process of metabolic diseases. Taken together, these findings provide new evidence and insights that XKY is a promising dietary formula in the treatment of metabolic disease by modulating hepatic glucolipid metabolism, the gut microbiota, and its metabolites in an in vivo model.

## Supplementary Information

**Additional file 1:** **FigureS1.** Comparison of the bacterial abundance in NC, Model andXKY-H groups. Data were shown as means ± SD. **FigureS2.** The relative abundance of L-Glutamine,L-Aspartic Acid, L-Arginine, N2-Acetyl-L-ornithine,L-Citrulline, N-Acetyl-L-glutamic acid2-Keto-glutaramicacid, L-Asparagine, Succinic acid,N-acetylaspartate, L-TryptophanL-Tyrosine,Chorismic acidMethoxyindoleacetic acidIndole-3-acetamide5-Hydroxy-L-tryptophanN'-FormylkynurenineIndoleSerotoninand 5-Hydroxyindolepyruvate. Data were shown asmeans ± SD. **Table S1.**List of primer sequences used for RT-PCR. **TableS2.** Chemicalcomposition identified from XKY in positive mode by UPLC-QE-MS analysis. **Table S3. **Chemicalcomposition identified from XKY in negative mode by UPLC-QE-MS analysis. 

## Data Availability

The datasets presented in this study can be found in online repositories. The original 16 S rDNA sequencing raw data were deposited into the NCBI database (Accession Number: PRJNA887907). The raw transcriptomic data were deposited into the NCBI database (Accession Number: PRJNA889248).

## Abbreviations

AAs: Amino acids
ALT: Alanine transaminase
AST: Aspartate aminotransferase
AUC: Area under the curve
BAs: Bile acids
BW: Body weight
Cyp51: Cytochrome P450, family 51
DCA: Deoxycholic acid
DEGs: Differential expression genes
Dhcr24: 24-dehydrocholesterol reductase
FBG: Fasting blood glucose
Fdps: Farnesyl diphosphate synthase
HFD: High-fat diet
HMDB: Human metabolome database
H&E: Hematoxylin and eosin
HOMA-IR: Homeostasis assessment of insulin resistance
Hmgcr: 3-hydroxy-3-methylglutaryl Coenzyme A reductase
Hmgcs: 3-hydroxy-3-methylglutaryl Coenzyme A synthase 1
Hsd17b7: 17 beta-hydroxysteroid dehydrogenase type 7
ITT: Insulin tolerance test
IHC: Immunohistochemical
Idi1: Isopentenyl-diphosphate delta isomerase 1
LCA: Lithocholic acid
LPS: Lipopolysaccharides
Lss: Lanosterol synthase
MET: Metformin
Mvd: Mevalonate diphosphate decarboxylase
Mvk: Mevalonate kinase
NAFLD: Non-alcoholic fatty liver disease
NC: Normal control group
OGTT: Oral glucose tolerance test
PAS: Periodic acid-schiff
SFAs: Short-chain fatty acids
QCs: Quality control samples
Sqle: Squalene epoxidase
TC: Total cholesterol
TCM: Traditional Chinese medicine
TG: Triglyceride
T2DM: Type 2 diabetes mellitus
XKY: Xiao-Ke-Yin
XKY-H: High dose of XKY
XKY-M: Middle dose of XKY
XKY-L: Low dose of XKY
