# Supplementary material for: Therapeutic mechanisms of the medicine and food homology formula Xiao-Ke-Yin on glucolipid metabolic dysfunction revealed by transcriptomics, metabolomics and microbiomics in mice
Source: Chin Med. 2023 May 18;18:57. doi: 10.1186/s13020-023-00752-6 (PMC10193706; doi:10.1186/s13020-023-00752-6)
Supplement: Supplementary file 1 — Additional file 1: FigureS1. Comparison of the bacterial abundance in NC, Model andXKY-H groups. Data were shown as means ± SD. FigureS2. The relative abundance of L-Glutamine,L-Aspartic Acid, L-Arginine, N2-Acetyl-L-ornithine,L-Citrulline, N-Acetyl-L-glutamic acid2-Keto-glutaramicacid, L-Asparagine, Succinic acid,N-acetylaspartate, L-TryptophanL-Tyrosine,Chorismic acidMethoxyindoleacetic acidIndole-3-acetamide5-Hydroxy-L-tryptophanN'-FormylkynurenineIndoleSerotoninand 5-Hydroxyindolepyruvate. Data were shown asmeans ± SD. Table S1.List of primer sequences used for RT-PCR. TableS2. Chemicalcomposition identified from XKY in positive mode by UPLC-QE-MS analysis. Table S3. Chemicalcomposition identified from XKY in negative mode by UPLC-QE-MS analysis. [file 13020_2023_752_MOESM1_ESM.docx]

Supplementary Material

**Supplementary Figures**


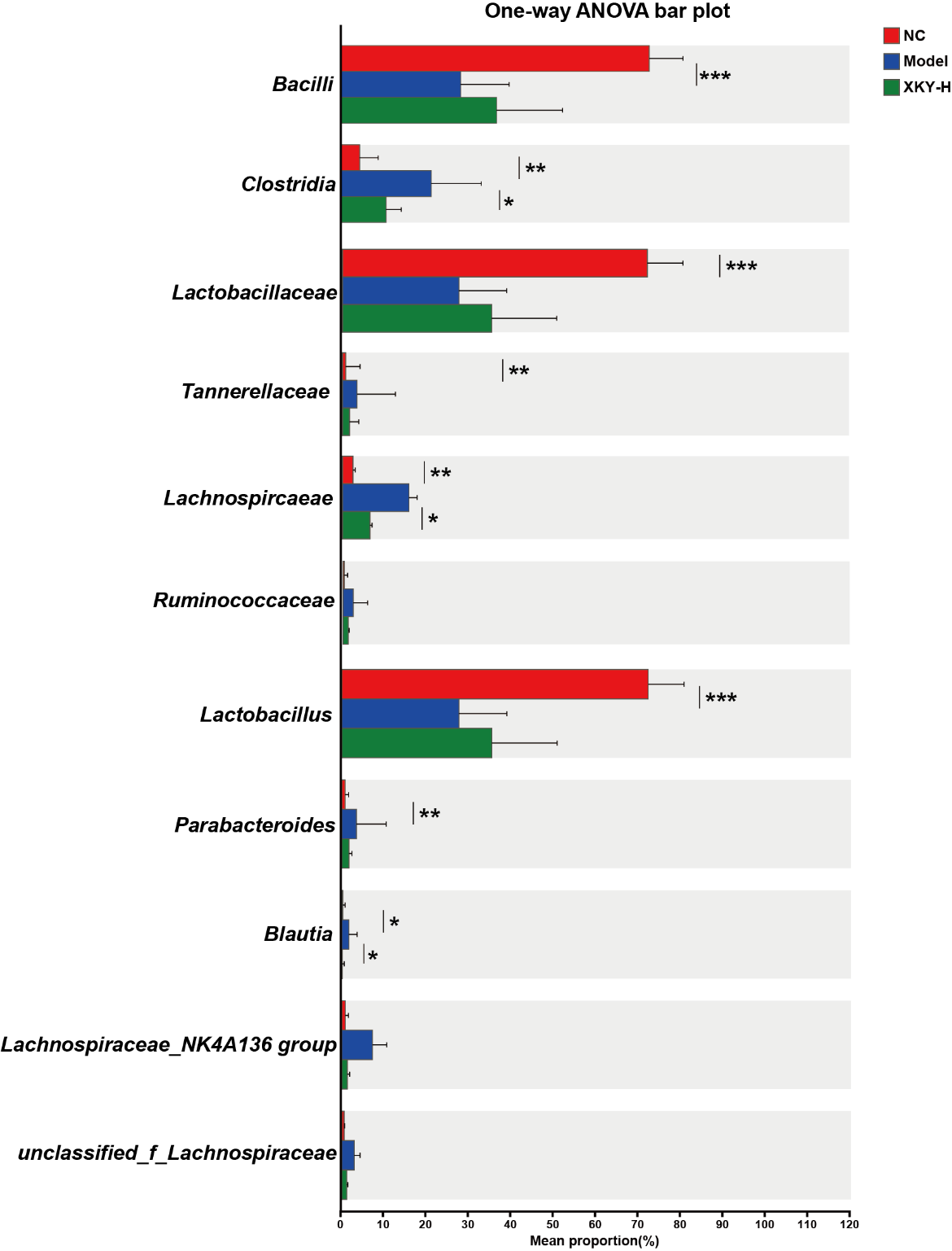


**Supplementary Figure 1.** Comparison of the bacterial abundance in NC, Model and XKY-H groups. Data were shown as means ± SD (n = 6, **P* < 0.05, ***P* < 0.01, ****P* < 0.001 vs. the Model group).

**
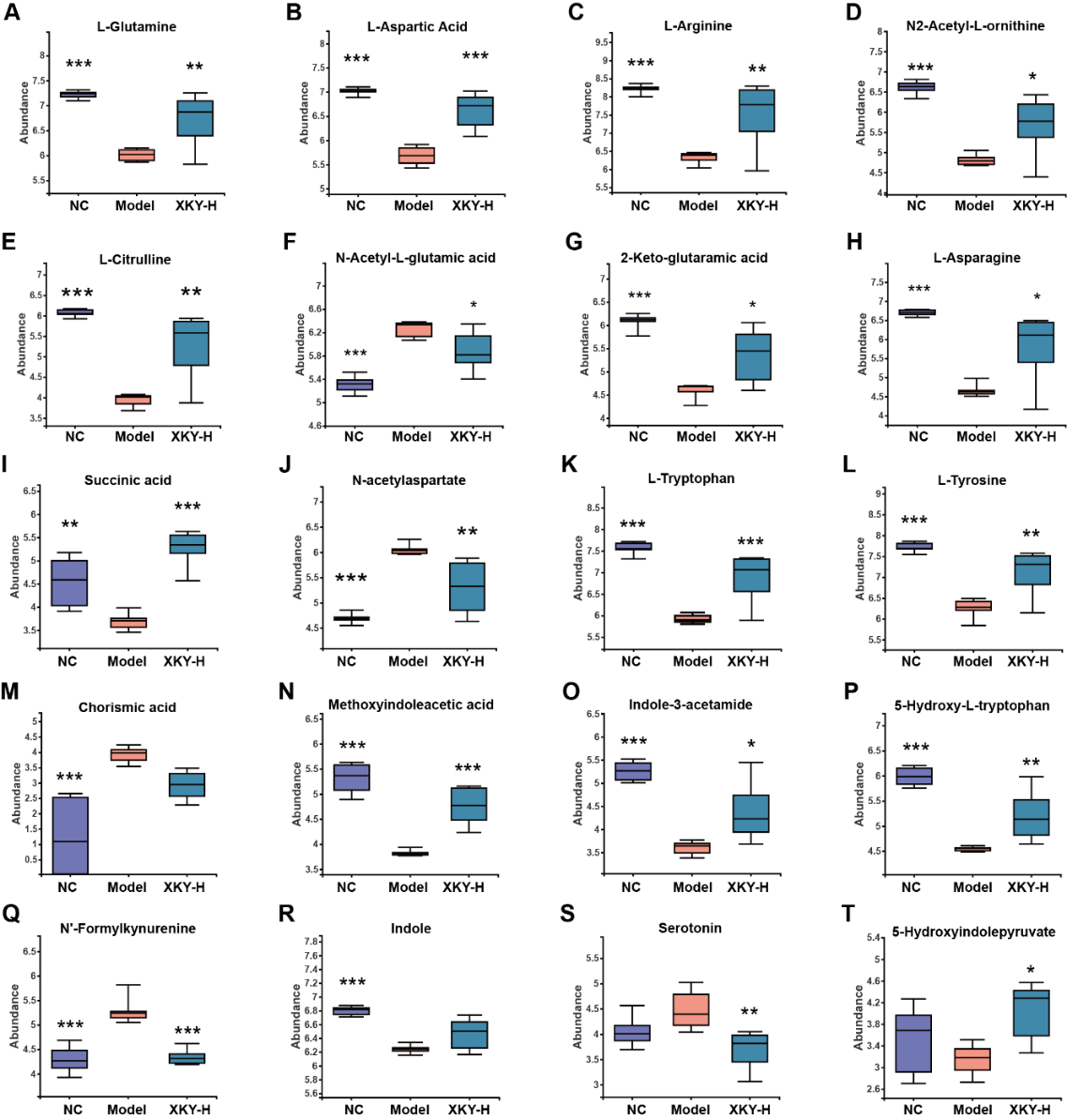
**

**Supplementary Figure 2.** The relative abundance of L-Glutamine (**A**), L-Aspartic Acid (**B**), L-Arginine (**C**), N2-Acetyl-L-ornithine (**D**), L-Citrulline (**E**), N-Acetyl-L-glutamic acid (**F**) 2-Keto-glutaramic acid (**G**), L-Asparagine (**H**), Succinic acid (**I**), N-acetylaspartate (**J**), L-Tryptophan (**K**) L-Tyrosine, (**L**) Chorismic acid (**M**) Methoxyindoleacetic acid (N) Indole-3-acetamide (**O**) 5-Hydroxy-L-tryptophan (**P**) N'-Formylkynurenine (**Q**) Indole (**R**) Serotonin (**S**) and 5-Hydroxyindolepyruvate (**T**). Data were shown as means ± SD (n = 6, **P* < 0.05, ***P* < 0.01, ****P* < 0.001 vs. the Model group).

**Supplementary Tables**

**Supplementary Table 1** List of primer sequences used for RT-PCR

| **Genes** | **Forward primer 5′>3′** | **Reverse primer 5′>3′** |
| --- | --- | --- |
| *Hmgcr* | CTTGTGGAATGCCTTGTGATTG | GAAGAATGTCATGAACACAAAGTAGTTG |
| *Mvk* | TCGGCCTCACACAAACTCTCT | CTGGCCAGGTGGTTCCTTAG |
| *Mvd* | CGGTCAACATCGCAGTTATCAA | GTGCAGCGTGACGCTCAG |
| *Idi1* | AAGCCGAGTTGGGAATACCCT | GTTCACCCCAGATACCATCAGATT |
| *Fdps* | TGTGTAGAACTGCTCCAGGCTTT | AAGCCTATGCCTGGCTTCTGA |
| *Sqle* | TTGTTGCGGATGGACTCTTCTCCA | GTTGACCAGAACAAGCTCCGCAAA |
| *Lss* | ATGAGTTGGGTCGGCAGAGAT | GCGCTTTTGGTAAGTCCGTG |
| *Cyp51* | GAGAGAAGTTTGCCTATGTGCC | TGTAACGGATTACTGGGTTTTCT |
| *Hsd17b7* | AGGCGTCGTGATGACCAATA | AAAAAGCGAAGGAGCCACATT |
| *Dhcr24* | GCACAGGCATCGAGTCATCAT | GTGCATCGCACAAAGCTGC |
| *Cyp7a1* | ACAGCTAAGGAGGACTTCACTCT | TTCATCAAGGTACCGGTCGTATT |
| *Cyp8b1* | CATGAAGGCTGTGCGTGAGGAA | CATCACGCTGTCCAACACTGGA |
| *Cyp27a1* | CCAGGCACAGGAGAGTACG | GGGCAAGTGCAGCACATAG |
| *Cyp7b1* | CCCTGCGTGACGAAATTGAC | AGAATAGTGCTTTCCAGGCAGA |
| *Claudin-1* | GGGGACAACATCGTGACCG | AGGAGTCGAAGACTTTGCACT |
| *ZO-1* | CTCAAGTTCCTGAAGCCCGT | GCAAAAGACCAACCGTCAGG |
| *FXR* | CCCCTGCTTGATGTGCTAC | CGTGGTGATGGTTGAATGTC |
| *FGF15* | ATGGCGAGAAAGTGGAACGG | CTGACACAGACTGGGATTGCT |
| *β-Actin* | CGTTGACATCCGTAAAGACCTC | TAGGAGCCAGGGCAGTAATCT |

**Supplementary Table 2** Chemical composition identified from XKY in positive mode by UPLC-QE-MS analysis

| **No.** | **Compound** | **Formula** | **Retention time (sec)** | **Response** | **Experimental (m/z)** | **Theoretical (m/z)** | **Mass accuracy (ppm)** | **Fragment ion (m/z)** |
| --- | --- | --- | --- | --- | --- | --- | --- | --- |
| POS 1 | Glucosamine | C_6_H_13_NO_5_ | 27.4750 | 2219273.5540 | 180.0866 | 180.0870 | 2.4109 | 293.182 70.065 114.102 |
| POS 2 | Arginine | C_6_H_14_N_4_O_2_ | 28.4623 | 917898656.4000 | 175.1187 | 175.1190 | 1.4322 | 543.132 |
| POS 3 | Adenine | C_5_H_5_N_5_ | 30.9349 | 125053338.6000 | 136.0617 | 136.0620 | 2.1236 | 108.044 126.054 |
| POS 4 | L-Valine | C_5_H_11_NO_2_ | 32.2848 | 971444045.4000 | 118.0860 | 118.0860 | 0.4193 | 144.101 |
| POS 5 | L-Proline | C_5_H_9_NO_2_ | 32.5416 | 43335900.0800 | 116.0705 | 116.0700 | 4.5056 | 254.101 |
| POS 6 | Myricetin | C_15_H_10_O_8_ | 33.1411 | 185413066.5000 | 319.0420 | 319.0420 | 0.0628 | 347.097 |
| POS 7 | Guanine | C_5_H_5_N_5_O | 33.5139 | 71040646.2200 | 152.0636 | 152.0570 | 43.3602 | 319.154 |
| POS 8 | L-Isoleucine | C_6_H_13_NO_2_ | 34.6520 | 38900494.4000 | 132.1018 | 132.1020 | 1.8266 | 216.123 234.132 188.092 |
| POS 9 | Tyrosine | C_9_H_11_NO_3_ | 36.6666 | 292795434.5000 | 182.0811 | 182.0810 | 0.7941 | 130.049 84.044 97.028 274.137 |
| POS 10 | Nicotinic acid | C_6_H_5_NO_2_ | 36.7626 | 239405400.4000 | 124.0392 | 124.0390 | 1.3294 | 194.08 104.158 86.059 |
| POS 11 | Phenethylamine | C_8_H_11_N | 46.2379 | 16071718.2800 | 122.0963 | 122.0960 | 2.6110 | 149.059 121.064 91.054 177.053 |
| POS 12 | Procyanidin B2 | C_30_H_26_O_12_ | 46.7345 | 7098812.7230 | 579.1496 | 579.1490 | 1.0770 | 455.215 437.204 |
| POS 13 | Epicatechin | C_15_H_14_O_6_ | 54.5380 | 7489652.9710 | 291.0863 | 291.0860 | 0.9991 | 196.132 108.044 126.054 |
| POS 14 | Geniposide | C_17_H_24_O_10_ | 64.7351 | 148287412.4000 | 427.0998 | 427.1000 | 0.5688 | 109.028 81.033 127.039 |
| POS 15 | Puerarin | C_21_H_20_O_9_ | 72.1034 | 204034317.0000 | 417.1180 | 417.1180 | 0.1024 | 149.059 227.079 121.064 163.076 |
| POS 16 | Caffeic acid | C_9_H_8_O_4_ | 76.2981 | 49640770.7500 | 181.0496 | 181.0500 | 2.1943 | 169.075 85.064 |
| POS 17 | 4-Hydroxybenzaldehyde | C_7_H_6_O_2_ | 81.8954 | 20339235.0800 | 123.0440 | 123.0440 | 0.3184 | 178.086 160.076 122.059 |
| POS 18 | 2-[[7-hydroxy-1-(4-hydroxy-3,5-dimethoxyphenyl)-3-(hydroxymethyl)-6,8-dimethoxy-1,2,3,4-tetrahydronaphthalen-2-yl]methoxy]-6-(hydroxymethyl)oxane-3,4,5-triol | C_28_H_38_O_13_ | 88.5064 | 1961202.1850 | 605.2203 | 605.2200 | 0.5612 | 360.104 300.087 342.098 |
| POS 19 | Hyperoside | C_21_H_20_O_12_ | 91.8952 | 8405243.2350 | 465.1028 | 465.1020 | 1.6776 | 99.044 71.049 127.038 |
| POS 20 | Methyl trans-cinnamic acid | C_10_H_10_O_2_ | 91.9419 | 25867915.7000 | 163.0754 | 163.0750 | 2.2677 | 99.044 71.049 127.038 |
| POS 21 | Vanillin | C_8_H_8_O_3_ | 94.4931 | 14282554.8900 | 153.0546 | 153.0550 | 2.3216 | 152.069 194.08 176.043 |
| POS 22 | Syringic acid | C_9_H_10_O_5_ | 98.9457 | 5948145.0530 | 199.0603 | 199.0600 | 1.4653 | 306.096 159.043 191.069 |
| POS 23 | Scopoletin | C_10_H_8_O_4_ | 99.9368 | 204067718.9000 | 193.0496 | 193.0500 | 1.8558 | 121.065 149.095 91.054 |
| POS 24 | Syringaldehyde | C_9_H_10_O_4_ | 100.3603 | 29001275.1200 | 183.0653 | 183.0650 | 1.3892 | 152.07 134.06 106.06 |
| POS 25 | 5-Hydroxymethylfurfural | C_6_H_6_O_3_ | 105.2040 | 318535384.3000 | 127.0389 | 127.0390 | 0.8518 | 166.048 138.054 234.076 140.034 |
| POS 26 | Loliolide | C_11_H_16_O_3_ | 109.2980 | 167421513.3000 | 197.1174 | 197.1170 | 2.0892 | 81.033 95.048 188.071 |
| POS 27 | Quercitrin | C_21_H_20_O_11_ | 117.5520 | 2256805.9680 | 449.1070 | 449.1070 | 0.1084 | 265.098 206.083 |
| POS 28 | Coumaric acid | C_9_H_8_O_3_ | 124.8030 | 91897464.8000 | 147.0442 | 147.0440 | 1.0577 | 81.033 218.081 |
| POS 29 | 3,5-Dicaffeoylquinic acid | C_25_H_24_O_12_ | 127.3065 | 1904057.5620 | 517.1337 | 517.1340 | 0.6249 | 188.071 160.076 81.033 |
| POS 30 | p-Coumaric acid | C_9_H_8_O_3_ | 140.8720 | 63596140.9800 | 165.0547 | 165.0550 | 1.6807 | 207.065 1890.53 161.059 175.039 |
| POS 31 | Benzoic acid | C_7_H_6_O_2_ | 144.1230 | 4994064.8820 | 123.0440 | 123.0440 | 0.3792 | 121.028 217.049 161.06 |
| POS 32 | Ferulic acid | C_10_H_10_O_4_ | 146.1755 | 12925754.5400 | 195.0663 | 195.0650 | 6.7183 | 170.08 142.087 98.059 164.069 |
| POS 33 | Sinapic acid | C_11_H_12_O_5_ | 160.0620 | 13523375.8000 | 225.0757 | 225.0760 | 1.5132 | 340.078 |
| POS 34 | 7,8-Dihydroxyflavone | C_15_H_10_O_4_ | 167.7155 | 737102449.9000 | 255.0646 | 255.0650 | 1.5227 | 99.043 121.966 71.048 |
| POS 35 | Ononin | C_22_H_22_O_9_ | 170.2615 | 15400883.2400 | 431.1328 | 431.1330 | 0.5179 | 218.21 |
| POS 36 | Luteolin | C_15_H_10_O_6_ | 184.0270 | 6559935.6630 | 287.0543 | 287.0540 | 1.1543 | 439.233 |
| POS 37 | Palmatine | C_21_H_22_NO_4_^+^ | 186.1515 | 1455822.4560 | 352.1541 | 352.1540 | 0.3788 | 218.081 200.07 81.033 |
| POS 38 | 4-Methylumbelliferone | C_10_H_8_O_3_ | 188.9220 | 32257449.3500 | 177.0548 | 177.0550 | 0.9963 | 189.053 95.012 161.06 119.049 |
| POS 39 | Formononetin-7-O-glucoside | C_22_H_22_O_9_ | 189.5895 | 9098264.4370 | 431.1353 | 431.1340 | 2.9729 | 166.086 87.043 108.044 136.075 |
| POS 40 | Glycitein | C_16_H_12_O_5_ | 197.6095 | 12360043.7200 | 307.0575 | 307.0570 | 1.6463 | 177.163 149.059 |
| POS 41 | Psoralen | C_11_H_6_O_3_ | 223.6250 | 236055.4956 | 187.0388 | 187.0390 | 0.8839 | 365.194 |
| POS 42 | Genistein | C_15_H_10_O_5_ | 223.8465 | 8767576.6810 | 271.0598 | 271.0600 | 0.6675 | 81.033 188.07 95.049 160.075 |
| POS 43 | Kaempferol | C_15_H_10_O_6_ | 232.5500 | 3340609.4130 | 287.0544 | 287.0550 | 2.1269 | 147.116 81.069 119.085 105.07 |
| POS 44 | Stevioside | C_38_H_60_O_18_ | 244.2380 | 5561159.0120 | 805.3837 | 805.3840 | 0.4109 | 255.064 |
| POS 45 | Camphor | C_10_H_16_O | 246.3310 | 4640348.4980 | 153.1272 | 153.1270 | 1.5830 | 245.118 247.143 275.125 |
| POS 46 | Scoparone | C_11_H_10_O_4_ | 279.1570 | 4753476.2330 | 207.0649 | 207.0650 | 0.2492 | 279.231 81.069 95.085 67.054 |
| POS 47 | Ligustilide | C_12_H_14_O_2_ | 294.9940 | 158475301.0000 | 191.1065 | 191.1070 | 2.4136 | 324.12 |
| POS 48 | (-)-Asarinin | C_20_H_18_O_6_ | 313.6310 | 15365055.4000 | 355.1152 | 355.1150 | 0.6314 | 349.198 |
| POS 49 | Biochanin A | C_16_H_12_O_5_ | 337.1785 | 6475871.1700 | 285.0754 | 285.0760 | 1.9680 | 353.23 |
| POS 50 | Chrysin | C_15_H_10_O_4_ | 340.2070 | 653088.0633 | 255.0650 | 255.0650 | 0.0275 | 81.069 93.07 67.054 195.138 |
| POS 51 | Crocetin | C_20_H_24_O_4_ | 361.2480 | 18203346.5900 | 329.1741 | 329.1740 | 0.1728 | 87.004 110.059 |
| POS 52 | Senkyunolide A | C_12_H_16_O_2_ | 377.8200 | 11422723.3900 | 193.1220 | 193.1220 | 0.0583 | 106.072 433.149 403.104 121.064 |
| POS 53 | 3-Butylidenephthalide | C_12_H_12_O_2_ | 455.4400 | 33989311.0800 | 189.0911 | 189.0910 | 0.3940 | 97.009 88.004 115.963 |
| POS 54 | (1R,2R,5S,8R,14R,15R,16S)-16-hydroxy-1,2,14,17,17-pentamethyl-8-(prop-1-en-2-yl)pentacyclo[11.7.0.0²,¹⁰.0⁵,⁹.0¹⁴,¹⁸]icosane-5,15-dicarboxylic acid | C_30_H_46_O_5_ | 497.5470 | 284395.4406 | 509.3237 | 509.3230 | 1.3644 | 207.102 115.964 189.09 133.974 |
| POS 55 | Ursolic acid | C_30_H_48_O_3_ | 507.5410 | 115964.6454 | 439.3562 | 439.3570 | 1.9294 | 87.004 110.06 113.963 96.009 |
| POS 56 | alpha-Linolenic acid | C_18_H_30_O_2_ | 561.7455 | 74729830.0700 | 279.2314 | 279.2320 | 2.0329 | 87.004 110.059 113.963 |
| POS 57 | Dibutylphthalate | C_16_H_22_O_4_ | 587.4640 | 21857434.5300 | 279.1591 | 279.1590 | 0.2335 | 320.254 |
| POS 58 | Choline [M]+ | C_5_H_14_NO | 671.6610 | 62054351.8400 | 104.1069 | 104.1070 | 1.3248 | 89.059 604.437 587.414 |
| POS 59 | Lupenone | C_30_H_48_O | 689.5170 | 556588.1645 | 425.3770 | 425.3770 | 0.0131 | 124.039 112.039 140.034 96.044 |
| POS 60 | Abietic acid | C_20_H_30_O_2_ | 695.2140 | 10681786.0400 | 303.2319 | 303.2320 | 0.3593 | 105.069 183.1 287.163 |
| POS 61 | (1S,2R,4aS,6aS,6bR,10S,12aR)-10-hydroxy-1,2,6a,6b,9,9,12a-heptamethyl-2,3,4,5,6,6a,7,8,8a,10,11,12,13,14b-tetradecahydro-1H-picene-4a-carboxylic acid | C_30_H_48_O_3_ | 696.3170 | 2835383.7310 | 457.3677 | 457.3670 | 1.4692 | 672.431 105.069 419.241 |
| POS 62 | Betaine | C_5_H_11_NO_2_ | 718.6020 | 144829228.4000 | 118.0863 | 118.0860 | 2.3887 | 57.033 69.033 73.028 |
| POS 63 | Linoleic acid | C_18_H_32_O_2_ | 732.7375 | 2592544.8030 | 281.2477 | 281.2480 | 1.1080 | 392.289 |
| POS 64 | Perillene | C_10_H_14_O | 745.2365 | 457134.9585 | 151.1117 | 151.1120 | 2.0062 | 754.381 |
| POS 65 | Di(2-ethylhexyl)phthalate (DEHP) | C_24_H_38_O_4_ | 777.3340 | 4553087.5080 | 391.2836 | 391.2840 | 1.0601 | 139.122 140.068 74.095 |

**Supplementary Table 3** Chemical composition identified from XKY in negative mode by UPLC-QE-MS analysis

| **No.** | **Compound** | **Formula** | **Retention time (sec)** | **Response** | **Experimental (m/z)** | **Theoretical (m/z)** | **Mass accuracy (ppm)** | | **Fragment ion (m/z)** |
| --- | --- | --- | --- | --- | --- | --- | --- | --- | --- |
| NEG 1 | Pyrogallol | C_6_H_6_O_3_ | 23.9009 | 4594412.1330 | 125.0244 | 125.0240 | 2.9263 | | 162.983 118.993 206.972 |
| NEG 2 | Allantoin | C_4_H_6_N_4_O_3_ | 31.1216 | 849514.6582 | 157.0366 | 157.0370 | 2.2556 | 89.024 251.075 | |
| NEG 3 | Fumaric acid | C_4_H_4_O_4_ | 31.83045 | 177378749.2000 | 115.0035 | 115.0040 | 4.4257 | 111.008 | |
| NEG 4 | Succinic acid | C_4_H_6_O_4_ | 33.99655 | 296991104.3000 | 117.0193 | 117.0190 | 2.6933 | 116.006 72.017 134.017 | |
| NEG 5 | L-Phenylalanine | C_9_H_11_NO_2_ | 39.2103 | 1174317.3340 | 164.0717 | 164.0720 | 1.9816 | 89.143 269.068 | |
| NEG 6 | Nicotinic acid | C_6_H_5_NO_2_ | 47.0939 | 45059148.4000 | 122.0247 | 122.0250 | 2.4240 | 585.154 89.024 | |
| NEG 7 | Amygdalin | C_20_H_27_NO_11_ | 56.6317 | 2777166.1400 | 456.1521 | 456.1510 | 2.4332 | 109.029 153.02 108.02 | |
| NEG 8 | Malic acid | C_4_H_6_O_5_ | 61.5542 | 84821809.3800 | 133.0141 | 133.0140 | 0.7164 | 121.029 | |
| NEG 9 | Geniposide | C_17_H_24_O_10_ | 63.0111 | 74514135.0800 | 387.1292 | 387.1300 | 2.1348 | 191.055 173.046 337.096 | |
| NEG 10 | Shanzhiside methyl ester | C_17_H_26_O_11_ | 69.0581 | 8391687.4820 | 405.1399 | 405.1400 | 0.1627 | 73.029 151.061 | |
| NEG 11 | Daidzin | C_21_H_20_O_9_ | 71.1478 | 89549236.7000 | 461.1099 | 461.1090 | 1.9461 | 123.045 95.014 124.04 | |
| NEG 12 | Gentisic acid | C_7_H_6_O_4_ | 72.9732 | 52300415.2300 | 153.0190 | 153.0190 | 0.1354 | 89.024 268.082 196.06 430.132 | |
| NEG 13 | 7,8-Dihydroxycoumarin | C_9_H_6_O_4_ | 77.65945 | 4520103.2710 | 177.0193 | 177.0190 | 1.4861 | 124.04 210.076 89.024 282.096 | |
| NEG 14 | p-Hydroxybenzaldehyde | C_7_H_6_O_2_ | 80.2075 | 78069859.4000 | 121.0294 | 121.0290 | 3.0894 | 89.024 376.171 59.013 | |
| NEG 15 | Coumaric acid | C_9_H_8_O_3_ | 85.6208 | 102253463.6000 | 163.0399 | 163.0400 | 0.8435 | 89.024 | |
| NEG 16 | Vanillic acid | C_8_H_8_O_4_ | 97.4898 | 7025804.5040 | 167.0347 | 167.0350 | 1.6713 | 449.201 269.139 59.013 287.15 | |
| NEG 17 | Taxifolin | C_15_H_12_O_7_ | 97.9356 | 10782959.3100 | 303.0507 | 303.0510 | 1.0382 | 160.841 89.024 59.013 85.029 | |
| NEG 18 | Rosmarinic acid | C_18_H_16_O_8_ | 101.5455 | 29856006.2000 | 359.0772 | 359.0770 | 0.6268 | 358.148 101.023 71.013 | |
| NEG 19 | Ferulic acid | C_10_H_10_O_4_ | 101.8405 | 534534080.3000 | 193.0506 | 193.0500 | 2.8658 | 89.024 | |
| NEG 20 | 4-Methylumbelliferone | C_10_H_8_O_3_ | 107.146 | 12247549.1500 | 175.0399 | 175.0400 | 0.4004 | 224.093 180.102 123.032 109.016 | |
| NEG 21 | Quercitrin | C_21_H_20_O_11_ | 112.827 | 4736803.4240 | 447.0939 | 447.0930 | 2.0550 | 223.062 163.04 91.055 161.06 | |
| NEG 22 | Citric acid | C_6_H_8_O_7_ | 118.001 | 25174859.8600 | 191.0194 | 191.0200 | 3.1113 | 132.867 115.003 71.014 | |
| NEG 23 | Genistein | C_15_H_10_O_5_ | 127.988 | 1776945.0880 | 269.0457 | 269.0450 | 2.7076 | 132.867 115.003 71.013 | |
| NEG 24 | Isochlorogenic acid B | C_25_H_24_O_12_ | 127.988 | 28558251.8000 | 515.1197 | 515.1200 | 0.5795 | 132.867 115.003 71.013 | |
| NEG 25 | Azelaic acid | C_9_H_16_O_4_ | 139.402 | 746787293.6000 | 187.0972 | 187.0970 | 1.3346 | 205.051 331.085 | |
| NEG 26 | 4-Hydroxybenzoic acid | C_7_H_6_O_3_ | 148.409 | 92778912.2700 | 137.0244 | 137.0240 | 2.5650 | 89.024 223.062 397.111 164.048 | |
| NEG 27 | Quercetin | C_15_H_10_O_7_ | 183.6655 | 4325172.4100 | 301.0357 | 301.0350 | 2.3653 | 233.067 89.024 279.087 | |
| NEG 28 | Isoimperatorin | C_16_H_14_O_4_ | 227.462 | 3092792.2510 | 269.0815 | 269.0820 | 1.7696 | 199.134 | |
| NEG 29 | Kaempferol | C_15_H_10_O_6_ | 232.162 | 3301296.0650 | 285.0401 | 285.0400 | 0.4250 | 134.864 135.97 | |
| NEG 30 | Diethyl-phthalate | C_12_H_14_O_4_ | 235.594 | 224325485.7000 | 221.0815 | 221.0820 | 2.3198 | 89.024 415.177 343.211 | |
| NEG 31 | Naringenin chalcone | C_15_H_12_O_5_ | 236.07 | 2233159.3990 | 271.0608 | 271.0610 | 0.6145 | 89.024 73.029 310.128 266.141 | |
| NEG 32 | Isorhamnetin | C_16_H_12_O_7_ | 245.494 | 10874849.8300 | 315.0508 | 315.0510 | 0.6064 | 134.864 135.97 | |
| NEG 33 | Apigenin | C_15_H_10_O_5_ | 267.021 | 1241780.7230 | 269.0457 | 269.0460 | 1.1013 | 329.231 223.059 195.064 | |
| NEG 34 | Crocin | C_44_H_64_O_24_ | 282.417 | 5048029.5390 | 975.3707 | 975.3710 | 0.2931 | 651.261 327.161 283.168 89.024 | |
| NEG 35 | Astragaloside IV | C_41_H_68_O_14_ | 326.3485 | 100687385.8000 | 829.4609 | 829.4590 | 2.2511 | 151.061 89.024 59.013 225.149 | |
| NEG 36 | Wogonin | C_16_H_12_O_5_ | 336.133 | 5922320.1720 | 283.0614 | 283.0610 | 1.5319 | 151.06 89.024 | |
| NEG 37 | Chrysin | C_15_H_10_O_4_ | 339.463 | 2474197.6770 | 253.0506 | 253.0510 | 1.6228 | 85.029 57.034 113.024 | |
| NEG 38 | Biochanin A | C_16_H_12_O_5_ | 360.385 | 1997612.3600 | 283.0616 | 283.0610 | 2.0191 | 861.44 | |
| NEG 39 | Astragaloside II | C_43_H_70_O_15_ | 405.5475 | 33438397.0400 | 871.4712 | 871.4700 | 1.4337 | 179.107 191.108 369.21 | |
| NEG 40 | Crocetin | C_20_H_24_O_4_ | 425.051 | 44420946.9000 | 327.1601 | 327.1600 | 0.1676 | 87.044 85.029 133.32 73.029 | |
| NEG 41 | Methyl hexadecanoate | C_17_H_34_O_2_ | 503.258 | 154813691.1000 | 315.2536 | 315.2540 | 1.3037 | 341.11 89.024 179.055 59.013 | |
| NEG 42 | Linolenic acid | C_18_H_30_O_2_ | 546.489 | 673783.9698 | 277.2170 | 277.2170 | 0.1333 | 112.986 | |
| NEG 43 | (1R,2R,5S,8R,14R,15R,16S)-16-hydroxy-1,2,14,17,17-pentamethyl-8-(prop-1-en-2-yl)pentacyclo[11.7.0.0²,¹⁰.0⁵,⁹.0¹⁴,¹⁸]icosane-5,15-dicarboxylic acid | C_30_H_46_O_5_ | 591.086 | 680866.2995 | 485.3282 | 485.3270 | 2.4901 | 59.013 134.988 71.013 89.024 | |
| NEG 44 | 9-HODE | C_18_H_32_O_3_ | 628.7145 | 8412504.2120 | 295.2280 | 295.2280 | 0.1341 | 255.231 483.275 152.995 | |
| NEG 45 | Sorbitol | C_6_H_14_O_6_ | 729.135 | 58093545.0700 | 181.0716 | 181.0720 | 2.2406 | 281.25 163.062 | |
| NEG 46 | Quinic acid | C_7_H_12_O_6_ | 732.125 | 38344124.6900 | 191.0561 | 191.0560 | 0.7264 | 341.11 89.024 59.013 179.055 | |
| NEG 47 | Gallic acid | C_7_H_6_O_5_ | 732.62 | 4555605.1960 | 169.0140 | 169.0140 | 0.2222 | 112.986 248.961 180.972 | |
| NEG 48 | Sucrose | C_12_H_22_O_11_ | 732.62 | 26047688.4100 | 341.1092 | 341.1090 | 0.7313 | 112.986 248.961 180.972 | |
| NEG 49 | Linoleic acid | C_18_H_32_O_2_ | 733.38 | 5391302.7780 | 279.2326 | 279.2330 | 1.5007 | 181.071 89.024 101.024 71.013 | |
| NEG 50 | Oleic acid | C_18_H_34_O_2_ | 757.655 | 3037113.4560 | 281.2490 | 281.2490 | 0.0128 | 381.172 116.928 99.925 | |
